# Fluorescent Protein Aided Insights on Plastids and their Extensions: A Critical Appraisal

**DOI:** 10.3389/fpls.2015.01253

**Published:** 2016-01-20

**Authors:** Kathleen Delfosse, Michael R. Wozny, Erica-Ashley Jaipargas, Kiah A. Barton, Cole Anderson, Jaideep Mathur

**Affiliations:** Laboratory of Plant Development and Interactions, Department of Molecular and Cellular Biology, University of GuelphGuelph, ON, Canada

**Keywords:** plastids, fluorescent proteins, photoconvertible fluorescent protein, stromules, stroma, retrograde signaling

## Abstract

Multi-colored fluorescent proteins targeted to plastids have provided new insights on the dynamic behavior of these organelles and their interactions with other cytoplasmic components and compartments. Sub-plastidic components such as thylakoids, stroma, the inner and outer membranes of the plastid envelope, nucleoids, plastoglobuli, and starch grains have been efficiently highlighted in living plant cells. In addition, stroma filled membrane extensions called stromules have drawn attention to the dynamic nature of the plastid and its interactions with the rest of the cell. Use of dual and triple fluorescent protein combinations has begun to reveal plastid interactions with mitochondria, the nucleus, the endoplasmic reticulum and F-actin and suggests integral roles of plastids in retrograde signaling, cell to cell communication as well as plant-pathogen interactions. While the rapid advances and insights achieved through fluorescent protein based research on plastids are commendable it is necessary to endorse meaningful observations but subject others to closer scrutiny. Here, in order to develop a better and more comprehensive understanding of plastids and their extensions we provide a critical appraisal of recent information that has been acquired using targeted fluorescent protein probes.

## Introduction

Plastids are organelles of purported endosymbiogenic origin characterized by the presence of multi-layered bounding membranes (Margulis, 1970; Hoober, 2007; Sato, 2007). Plastids with an inner and an outer bounding membrane are accepted as a defining feature of plants and green algae (Wise, 2007; Pyke, 2009). Publications on plastids and the fundamentals of our present knowledge on these organelles are traceable to the late seventeenth century (Leeuwenhoek, 1674; reviewed by Gunning et al., 2007). A paradigm shift in plastid biology came with the realization that irrespective of their wide diversity of form and function, all plastid types are inter-convertible and are derived from colorless pro-plastids (Schmidt, 1870; Schimper, 1882). A classification based upon internal pigmentation was suggested (Schimper, 1882, 1883; Senn, 1908) and is followed even today; Accordingly plastids containing green pigment (chlorophyll) are called chloroplasts, plastids with other colored pigments are considered chromoplasts and colorless plastids are called leucoplasts.

Light microscopy observations and transmission electron microscopy (TEM) further established the presence of internal membranes in all plastids. The flattened membrane sacs were named thylakoids (Menke, 1962) and their stacking into prominent grana accounts for the characteristic lens shaped plastid body of chloroplasts. Relatively less organized pro-lamellar bodies and scattered thylakoids account for the lack of a well-defined plastid body and the elongated and pleomorphic leucoplasts and etioplasts (Gunning, 2001; Wise, 2007). Thylakoids in all plastid types are surrounded by a fluid stroma. Plastids also possess their own DNA condensed within nucleoids as well as protein translation machinery. Since different plastids synthesize and accumulate starches, lipids, oils and proteins they are further sub-classified on the basis of their major content and function (Wise, 2007). Despite the diversity of form and function the plastid unit is circumscribed by the double membrane-envelope.

**Figure 1:**
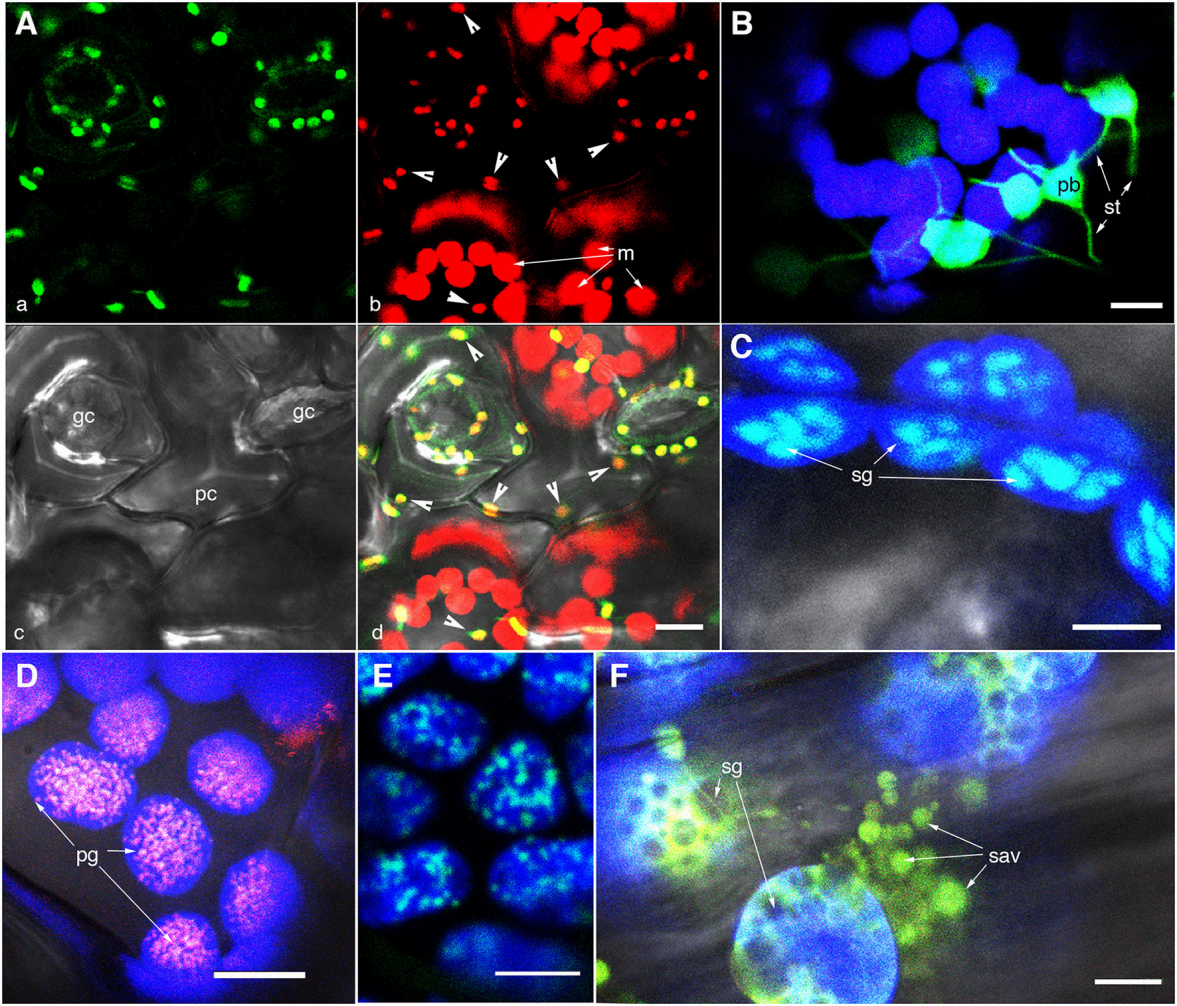
**Representative images of fluorescently highlighted plastids and some sub-plastidic features**. **(A)** A top-down view of epidermal and mesophyll chloroplasts in the upper epidermis of a green house grown Arabidopsis plant expressing the stroma-targeted tpFNR:GFP. Panel “a” shows the green fluorescent stroma (488 nm excitation; emission collected—509–520 nm). Panel “b” shows chlorophyll fluorescence in red (emission band 650–750 nm) in guard cells (gc), pavement cells (pc; arrowheads in b,d), and mesophyll cell (m) chloroplasts. Note the difference in size and the GFP signal intensity between the epidermal and mesophyll chloroplasts. **(B)** A view of thin stroma-filled tubules (stromules; st) and the bulky, grana-containing plastid body (pb) in epidermal chloroplasts of tobacco. **(C)** Starch grains (sg) in mesophyll chloroplasts highlighted in an Arabidopsis plant expressing a granule bound starch synthase (GBSS) fused to GFP. **(D)** Clusters of plastoglobuli (pg) observed in senescent leaves of Arabidopsis expressing a Fibrillin4:mEosFP fusion. **(E)** The highlighting of nucleoids in chloroplasts is indicated in a transgenic Arabidopsis plant expressing a plastid envelope DNA-binding (PEND) GFP fusion. **(F)** View of gerontoplasts in senescent leaves in an Arabidopsis plant expressing stroma-targeted tpFNR:GFP shows their swollen appearance suggesting compromised envelope membranes, degrading chlorophyll, the presence of starch grains (sg)visible as dark non-fluorescent regions and clusters of senescence associated vesicles (sav) containing fluorescently GFP-labeled storm content. Chlorophyll auto-fluorescence in **(B–F)** is false colored blue. Size bars = 5 μm in **(B,C)**; 10 μm in **(A,D,E,F)**.

Although transmission electron micrographs form the basis for our understanding of plastid ultrastructure, an appreciation of the dynamic nature of these fundamental organelles developed has with the advent of time-lapse imaging and cinephotomicrographic techniques (Wildman et al., 1962; Green, 1964; Menzel, 1994; Gunning, 2005). Whereas chloroplasts display strong auto-fluorescence (Figure 1A) and can therefore be easily identified under ultra-violet and blue light excitation, many more insights on plastids have come through the discovery of GFP and its potential as a fluorescent probe for living cells (Chalfie et al., 1994). Now, after more than 20 years of fluorescent protein (FP) aided research a large number of protein fusions have highlighted plastids and sub-plastidic structures as well as transient metabolites such as starches and lipids (Table 1; Figure 1). The use of double and triple transgenic plants has also facilitated observations on plastid interactions with other cellular components (Kwok and Hanson, 2003, 2004a,b; Schattat et al., 2011a). However, in comparison to conventional botanical micro-techniques and TEM where chemical fixation ensure that the cells and tissue do not change during observations living plant cells continue responding even as they are being observed. While every new publication underscores the tremendous potential of the FP-based approach for increasing insights on plastids it is also equally apparent that many artifacts are being reported and perpetuated. The situation becomes quite problematic when multiple reviews and follow-up publications strengthen a particular viewpoint and require considerably more effort for reevaluation of the original observations. This critical appraisal applauds the considerable insights on plastids obtained to date through the use of plastid-targeted FPs. It also points to the pitfalls and in some cases suggests alternative explanations that might be useful in furthering our knowledge on these essential organelles of the plant cell.

**Table 1:** **A non-comprehensive list of fluorescent proteins targeted to plastids**.

**Localization**	**Gene**	**FP**	**Organism of expression**	**T/P**	**References**
Stroma	TP-RecA	G	Petunia and *N. tabacum*	P	Köhler et al., 1997
	TP-*ent*-kaurene synthase (TP-AtKS1)	G	*N. tabacum*	T	Helliwell et al., 2001
	TP-*ent*-kaurene oxidase (TP-AtKO1)	G	*N. tabacum*	T	Helliwell et al., 2001
	TP-copalyl diphosphate synthase (TP-AtCPS1)	G	*N. tabacum*	T	Helliwell et al., 2001
	TP-small subunit of ribulose 1,5 bisphospate carboxylase (TP-RbcS)	G	*N. tabacum*	T	Helliwell et al., 2001
	Acyl-carrier protein (ACP)	R	*A. cepa*	T	Schnurr et al., 2002
	TP-ferredoxin NADP(H) oxidoreductase (TP-FNR)	G	*A. thaliana*	P	Marques et al., 2004
	TP-Plastocyanin (TP-PC)	G	*A. thaliana*	P	Marques et al., 2004
	TP-33kDa subunit of the oxygen evolving system of photosystem II (TP-PSII-O)	G	*A. thaliana*	P	Marques et al., 2004
	TP-ferredoxin NADP(H) oxidoreductase (TP-FNR)	E	*N. benthamiana, A. thaliana*	T/P	Schattat et al., 2012a
	Small subunit of ribulose 1,5 bisphospate carboxylase (SSU)	G	*A. thaliana*	P	Kim and Apel, 2004
	NADPH-dependent protochlorophyllide oxidoreductase A (PORA)	G	*A. thaliana*	P	Kim and Apel, 2004
	Thylakoid formation 1 (THF1)	G	*A. thaliana*	P	Wang et al., 2004
	Aspartate aminotransferase 5 (ASP5)	G	*N. tabacum*	P	Kwok and Hanson, 2004a
	Small subunit 3A of ribulose 1,5 bisphospate carboxylase (RbcS-3A)	C	*N. tabacum*	P	Kwok and Hanson, 2004a
	TP- Small subunit 3A of ribulose 1,5 bisphospate carboxylase (TP-RbcS-3A)	C	*N. tabacum*	P	Kwok and Hanson, 2004a
	α–carbonic anhydrase (CAH1)	G	*A. thaliana*	T	Villarejo et al., 2005
	Snowy cotyledon 1 (SCO1)	G	*A. thaliana*	P	Albrecht et al., 2006
	Allene oxide cyclase (AOC)	G	*A. thaliana, S. tuberosum* cv. Desiree	T/P	Farmaki et al., 2007
	Mesophyll-cell RNAi library line 7 –like (MRL7-L)	G	*N tabacum*	T	Qiao et al., 2011
	Accumulation and Replication of Chloroplasts 3 (ARC3)	Y	*N. tabacum*	T	Maple et al., 2007
	Chloroplast sensor kinase (CSK)	G	*N. tabacum*	T	Puthiyaveetil et al., 2008
	ADP-sugar pyrophosphatase (StASPP)	G	*A. thaliana, S. tuberosum*	P	Muñoz et al., 2008
	TP-Spo0B GTP-binding protein like (TP-AtOBGL)	G	*N. tabacum*	T	Chigri et al., 2009
	TP-Granule bound starch synthase I (TP-GBSSI)	Y	*T. aestivum L*	P	Shaw and Gray, 2011
	Starch synthase 1 (SS1)	G	*N. benthamiana*	T	Gámez-Arjona et al., 2014a
	3-ketoacyl-ACP reductase (KAR)	G	*P. patens*	T	Mueller et al., 2014
	PeroxiredoxinQ A (PrxQA)	G	*P. patens*	T	Mueller et al., 2014
Outer envelope	Outer envelope membrane protein 7 (AtOEP7)	G	*A. thaliana*	T/P	Lee et al., 2001
	GTP-Binding domain of AtToc159 (AtToc159G)	G	*A. thaliana*	T	Bauer et al., 2002
	Long-chain acyl-CoA synthetase 9 (LACS9)	G	*A. cepa*	T	Schnurr et al., 2002
	Crumpled leaf (CRL)	G	*A. thaliana*	P	Asano et al., 2004
	Chloroplast unusual positioning 1 (CHUP1)	G	*A. thaliana*	P	Oikawa et al., 2008
	Sensitive to freezing 2 (SFR2)	C	*A thaliana*	P	Ferro et al., 2010
	Translocon at the outer membrane of chloroplasts 64 (AtTOC64)	G	*N. benthamiana*	T	Breuers et al., 2012
Inner envelope	Monogalactosyldiacylglycerol synthase 1 (MGD1)	G	*A. thaliana*	T	Awai et al., 2001
	Inner envelope protein 60 (IEP60)	G	*A. thaliana*	T	Ferro et al., 2002
	Chloroplast envelope quinone oxidoreductase homolog (ceQORH)	G	*A. thaliana, N. Tabacum*	T	Miras et al., 2002
	Triose phosphate translocator (AtTPT)	G	*N. benthamiana*	T	Breuers et al., 2012
	Albino or pale green mutant 1 (AtAPG1)	G	*N. benthamiana*	T	Breuers et al., 2012
	Giant Chloroplast 1 (GC1)	Y	*A. thaliana*	P	Maple et al., 2004
	Chloroplast import apparatus 5 TP and first 2 transmembrane domains (prCIA5TP-TM2)	R	*A. thaliana*	T	Teng et al., 2006
	Translocon at the inner envelope membrane of chloroplasts 40 (Tic 40)	Y	*A. thaliana*	T	Bédard et al., 2007
	Translocon at the inner envelope membrane of chloroplasts 110 (Tic110)	Y	*A. thaliana*	T	Bédard et al., 2007
	Translocon at the inner envelope membrane of chloroplasts 20 I (TIC20-I)	Y	*A. thaliana*	T	Kasmati et al., 2011
	Translocon at the inner envelope membrane of chloroplasts 20 II (TIC20-II)	Y	*A. thaliana*	T	Kasmati et al., 2011
	Translocon at the inner envelope membrane of chloroplasts Tic20 IV (TIC20-IV)	Y	*A. thaliana*	T	Kasmati et al., 2011
	Translocon at the inner envelope membrane of chloroplasts Tic20 V (TIC20-V)	Y	*A. thaliana*	T	Kasmati et al., 2011
	Translocon at the inner membrane of chloroplasts 21 (TIC21)	Y	*A. thaliana*	T	Yang et al., 2012
	AtLrgB	G	*A. thaliana*	P	Yang et al., 2012
Thylakoid	Sulfurtransferase 15 (AtSTR15)	G	*A. thaliana*	T	Bauer et al., 2004
	NADPH-dependent protochlorophyllide oxidoreductase B (PORB)	G	*A. thaliana*	P	Kim and Apel, 2004
	N-terminal region of P-type ATPase of Arabidopsis 2 (PAA2)	G	*A. thaliana*	T	Abdel-Ghany et al., 2005
	Allene oxide synthase 1 (AOS1)	G	*A. thaliana*	T	Farmaki et al., 2007
	Allene oxide synthase 2 (AOS2)	G	*A. thaliana*	T	Farmaki et al., 2007
	Hydroperoxide lyase (HPL)	G	*A. thaliana*	T	Farmaki et al., 2007
	Chlorophyll A/B binding protein 180 (CAB180)	G	*A. thaliana*	T	Farmaki et al., 2007
	FE superoxide dismutase 2 (FSD2)	G	*N. tabacum*	T	Myouga et al., 2008
	High chlorophyll fluorescence 106 (Hcf106)	G	*N. tabacum*	T	Vladimirou et al., 2009
	Thylakoid soluble phosphoprotein (AtTSP9)	C	*A. thaliana*	P	Ferro et al., 2010
	Curvature thylakoid 1A (CURT1A)	R	*A. thaliana*	T	Armbruster et al., 2013
	Curvature thylakoid 1B (CURT1B)	R	*A. thaliana*	T	Armbruster et al., 2013
	Curvature thylakoid 1D (CURT1D)	R	*A. thaliana*	T	Armbruster et al., 2013
	Starch synthase 4 (SS4)	G	*N. benthamiana*	T	Gámez-Arjona et al., 2014a
	TP-16kDa subunit of the oxygen evolving system of photosystem II (TP-PSII-Q)	G	*A. thaliana*	P	Marques et al., 2004
	TP-23kDa subunit of the oxygen evolving system of photosystem II (TP-PSII-P)	G	*A. thaliana*	P	Marques et al., 2004
Starch granule	Granule bound starch synthase (GBSS)	G	*A. thaliana*	P	Szydlowski et al., 2009; Bahaji et al., 2011
	Dual-specificity protein phosphatase 4 (DSP4)	G	*A. thaliana*	P	Sokolov et al., 2006
	Isoamylase 3 (ISA3)	G	*A. thaliana*	T	Delatte et al., 2006
	Starch binding domain of Glucan, water dikinase 3 (GWD3-SBD)	Y	*N. benthamiana*	T	Christiansen et al., 2009
	Like SEX4 1 (LSF1)	G	*N. benthamiana*	T	Comparot-Moss et al., 2010
Plastoglobules	Plastoglobulin 30.4 (AtPGL30.4)	G	*A. thaliana*	T	Vidi et al., 2006
	Plastoglobulin 34 (AtPGL34)	G	*A. thaliana*	T	Vidi et al., 2006
	Plastoglobulin (AtPGL35)	G	*A. thaliana*	T	Vidi et al., 2006
	Fructose-1,6,-bisphosphate aldolase 1 (AtFBA1)	G	*A. thaliana*	T	Vidi et al., 2006
	Fructose-1,6-bisphosphate aldolase 2 (AtFBA2)	G	*A. thaliana*	T	Vidi et al., 2006
	Tocopherol cyclase 1 (AtVTE1)	Y	*A. thaliana*	T	Vidi et al., 2006
	NAD(P)H dehydrogenase C1 (NDC1)	Y	*N. benthamiana*	T	Piller et al., 2011
	Phytoene synthase (AtPSY)	R	*V. unguiculata* subsp. *unguiculata*	T	Shumskaya et al., 2012
	Phytoene synthase 1 (OsPSY1)	G	*Z. Mays*	T	Shumskaya et al., 2012
	Phytoene synthase 2 (OsPSY2)	G	*Z. Mays*	T	Shumskaya et al., 2012
	Phytoene synthase 3 (OsPSY3)	G	*Z. Mays*	T	Shumskaya et al., 2012
	Phytoene synthase 2 (ZmPSY2)	G	*Z. Mays*	T	Shumskaya et al., 2012
	Phytoene synthase 3 (ZmPSY3)	G	*Z. Mays*	T	Shumskaya et al., 2012
	Plastoglobulin 2 (ZmPG2)	R	*Z. Mays*	T	Shumskaya et al., 2012
	Fibrillin 1b (FBN1b)	G	*N. benthamiana*	T	Gámez-Arjona et al., 2014b
Nucleoids	N-terminus of Plastid envelope DNA binding (PEND)	G	*A. thaliana*	P	Terasawa and Sato, 2005
	Apurinic endonuclease-redox protein (ARP)	G	*A. thaliana*	T	Gutman and Niyogi, 2009
	Endonuclease three homolog 1 (AtNTH1)	G	*A. thaliana*	T	Gutman and Niyogi, 2009
	Endonuclease three homolog 2 (AtNTH2)	G	*A. thaliana*	T	Gutman and Niyogi, 2009
	Fructokinase-like (FLN1)	Y	*N. tabacum*	T	Arsova et al., 2010
	Fructokinase-like (FLN2)	Y	*N. tabacum*	T	Arsova et al., 2010
	Mesophyll-cell RNAi library line 7 (MRL7)	G	*N. tabacum*	T	Qiao et al., 2011
	Plastid transcriptionally active chromosome 3 (pTAC3)	G	*A. thaliana*	T	Yagi et al., 2012
	Lac repressor (Lacl)	G	*N. tabacum*	P	Newell et al., 2012
	SWIB domain containing protein 2 (SWIB-2)	G	*N. tabacum*	T	Melonek et al., 2012
	SWIB domain containing protein 3 (SWIB-3)	G	*N. tabacum*	T	Melonek et al., 2012
	SWIB domain containing protein 4 (SWIB-4)	G/R	*N. tabacum*	T	Melonek et al., 2012
	SWIB domain containing protein 6 (SWIB-6)	G/R	*N. tabacum*	T	Melonek et al., 2012

Plant species: Triticum aestivum L.; Arabidopsis thaliana; Nicotiana benthamiana/tabacum; Solanum tuberosum; Zea mays; Allium cepa; Physcomitrella patens. FP, Fluorescent Protein; E, mEosFP; G, GFP; R, RFP; Y, YFP; P, Transgenic Plant; T, Transient expression; TP, Transit Peptide/presequence.

With the exception of the TP-GBSS driven under the Rice Act1 promoter and the LacI plastid nucleoid probe driven by a tobacco psbA gene all other probes reported here used the Cauliflower Mosaic Virus 35S promoter.

## Stroma-targeted FPs have led to major insights concerning the dynamic nature of plastids

The targeting of a GFP to the stroma (Köhler et al., 1997) was one of the earliest successful demonstrations of the use of FP-technology for understanding plastids. Earlier light microscopy based investigations had already established the dynamic behavior of plastids in response to light and other environmental factors, now considered as text-book information (Pyke, 2009; Buchanan et al., 2015; Taiz et al., 2015). Differential interference contrast (DIC) cinephotomicrography of chloroplasts suggested an undulating envelope that was likened to a mobile jacket surrounding the plastid body (Wildman et al., 1962). Stroma-targeted GFP confirmed the earlier observations and highlighted thin stroma filled tubules, subsequently named stromules, that extended and retracted in relation to the main chloroplast body (Köhler et al., 1997; Köhler and Hanson, 2000; Figure 1B). The excitement generated by this seminal discovery led several groups to start generating fusing proteins (Tirlapur et al., 1999; Arimura et al., 2001; reviewed by Natesan et al., 2005) that could highlight stromules and allow investigations on the conditions that promote or repress stromule formation and can provide insights into their function. In general in plants stably expressing stroma-targeted FPs the epidermal plastids appear more fluorescent as compared to mesophyll chloroplasts. This has led to an erroneous impression in the mind of the non-specialist that mesophyll chloroplasts do not exhibit stromules while the most extensive and numerous stromules are observed in non-green plastids (Köhler and Hanson, 2000). Stromule formation has been observed in response to alteration in plastid redox status (Itoh et al., 2010; Brunkard et al., 2015), elevated temperatures (Holzinger et al., 2007a), symbiotic interactions (Fester et al., 2001; Hans et al., 2004; Lohse et al., 2005), virus and bacterial infection (Caplan et al., 2008; Krenz et al., 2012, 2014; Erickson et al., 2014) and growth regulator and mineral nutrient stress (Gray et al., 2012; Glińska et al., 2015). Stromule formation is also attributed to changes in plastid size and density within a cell (Pyke and Howells, 2002; Waters et al., 2004). As observations on stromules and changes in plastid morphology increase the fresh insights and opinions resulting from them are discussed in more detail.

## Diurnal changes in plastid morphology

Stroma-targeted FPs made it easier to follow plastid behavior in real time under different physiological states of the plant cell. It was found that the morphology of plastids changed considerably during the day–night cycle. The frequency of stromule formation from plastids increased during daytime and reverted to a low, basal frequency at night (Schattat et al., 2011a; Brunkard et al., 2015). A clear link to photosynthesis and sucrose production was suggested by this diurnal phenomenon (Schattat and Klösgen, 2011). This was confirmed through exogenous sucrose feeding which also increased the frequency of stromule formation (Schattat and Klösgen, 2011; Schattat et al., 2012a). Notably, other conditions such as pathogen infection (Fester et al., 2001; Lohse et al., 2005; Krenz et al., 2010, 2012; Erickson et al., 2014; Caplan et al., 2015) and senescence (Ishida et al., 2008), that affect the sugar status of a plant cell also increase stromule frequency. Whereas sugar appears to be a universal signal for changes in plastid morphology a recent report (Brunkard et al., 2015) suggests that changes in the internal redox status of chloroplasts, which precede the production of photosynthates, are responsible for stromule formation.

The conclusion that light-sensitive redox signals triggered within chloroplasts play a major role in stromule formation are based on the use of DCMU and DBMIB, two chemical inhibitors of the photosynthetic electron transport chain (pETC) (Brunkard et al., 2015). It was observed that treatment of 14-day old excised cotyledons of *Nicotiana benthamiana* and *Arabidopsis thaliana* for 2 h with these inhibitors resulted in a significant increase in stromule frequency of chloroplasts. The presence of chloroplasts was demonstrated in pavement and guard cells in the tobacco epidermis (Dupree et al., 1991) and the researchers found increased stromule frequency in both cell types (Brunkard et al., 2015). However, the increase in stromules was limited to only guard cells and not observed in the pavement cells of Arabidopsis. In order to explain the absence of stromules in Arabidopsis cotyledon pavement cells an unreferenced statement—“unlike *N. benthamiana*, the epidermis of *A. thaliana* has two distinct types of plastids: chloroplasts in the guard cells and leucoplasts in the pavement cells,” was presented (Brunkard et al., 2015). A diagrammatic depiction of this statement was used to present a model where reactive oxygen species (ROS) generated from the pETC triggers stromule formation in chloroplasts but sucrose produced by chloroplasts in the mesophyll layer is responsible for stromules in the so-called pavement cell leucoplasts (Brunkard et al., 2015). Interestingly a number of publications actually document the presence of chloroplasts in epidermal pavement cells in Arabidopsis (Robertson et al., 1996; Vitha et al., 2001; Joo et al., 2005). An authoritative book on plastid biology (Pyke, 2009) provides the unambiguous statement—“in many texts, it is stated that epidermal cells lack chloroplasts, which is untrue.” It is also noteworthy that the major conclusions of Brunkard et al. (2015) are based on observations of excised cotyledons and not true, photosynthesizing leaves. Plastids in wounded as well as senescent tissue are known to show increased stromule frequency (Krupinska, 2007; Ishida et al., 2008). We conclude that the model presented by Brunkard et al. (2015) suggesting change in internal chloroplast redox as a trigger for stromule formation, even though based on an assumption of leucoplasts in Arabidopsis pavement cells, is very interesting and requires further critical evaluation.

## Chloroplast protrusions and stromules: an artificial distinction?

During recent years FP-highlighted plastids and stromules have garnered a fair bit of attention but another contemporary undercurrent of contextual publications based on TEM studies has also existed and requires discussion. Several publications that predate the discovery and naming of stromules, presented double membrane bound stroma-filled protrusions that were simply called chloroplast protrusions (CP) (Bonzi and Fabbri, 1975; Lütz and Moser, 1977; Lütz, 1987; Bourett et al., 1999). Serial TEM sections of leaves in *Ranunculus glacialis* and *O. digyna* (Lütz and Moser, 1977; Lütz, 1987; Larcher et al., 1997; Lütz and Engel, 2007) showed that CP appear as broad or long, grana-free extensions and occasionally form pocket-like structures with mitochondria and microbody aggregates (Lütz and Engel, 2007). While the underlying basis for the statement is unclear researchers on CP appear to have distanced themselves from observations of stromules by declaring that CP and stromules are different (Buchner et al., 2007a,b, 2013, 2014; Holzinger et al., 2007b; Lütz and Engel, 2007; Lütz et al., 2012; Moser et al., 2015). An appraisal of the publications suggests that the only difference is that as compared to CP observed in electron micrographs the stromules are very thin, with diameters less than 800 nm and up to 50 μm long (Köhler and Hanson, 2000). However, emphasis on the thinness of the stromule was made in order to differentiate them from the generally flexible non-photosynthetic plastids that appear irregularly shaped, amoeboid, round to oblong to elongated and form lobes, knobs and loops (Köhler et al., 1997; Köhler and Hanson, 2000; Kwok and Hanson, 2004d). While discussing the early studies in relation to the paucity of electron micrographs of stromules it was pointed out that studies on CP focused on the leaf tissue, in which stromules are not common, and that stromules are not well preserved by standard fixation methods for electron microscopy (Köhler and Hanson, 2000). Today both statements cannot be upheld since numerous observations on stromules in leaf tissue have been published at both the light microscopy and TEM level (Holzinger et al., 2008; Sage and Sage, 2009; Schattat et al., 2012a). A major effort was made to figure out clear differences between the two sets of observations by Holzinger et al. (2007b) by creating a “shape index” to compare the different sizes and volumes of stromules with those of temperature-induced protrusions in *A.thaliana*. Interestingly this study concedes that “an interchange between these groups might still be possible,” and whether a protrusion goes on to become a stromule of more typical length and diameter might depend on the sub-cellular space available and the unknown factors that cause stromule growth (Holzinger et al., 2007b). Equally interesting is a contextual comprehensive review that cites the Holzinger et al. (2007b) publication as strong evidence of differences between CP and stromules but also presents a table that lists Arabidopsis as a plant that does not produce CP (Lütz, 2010).

**Figure 2:**
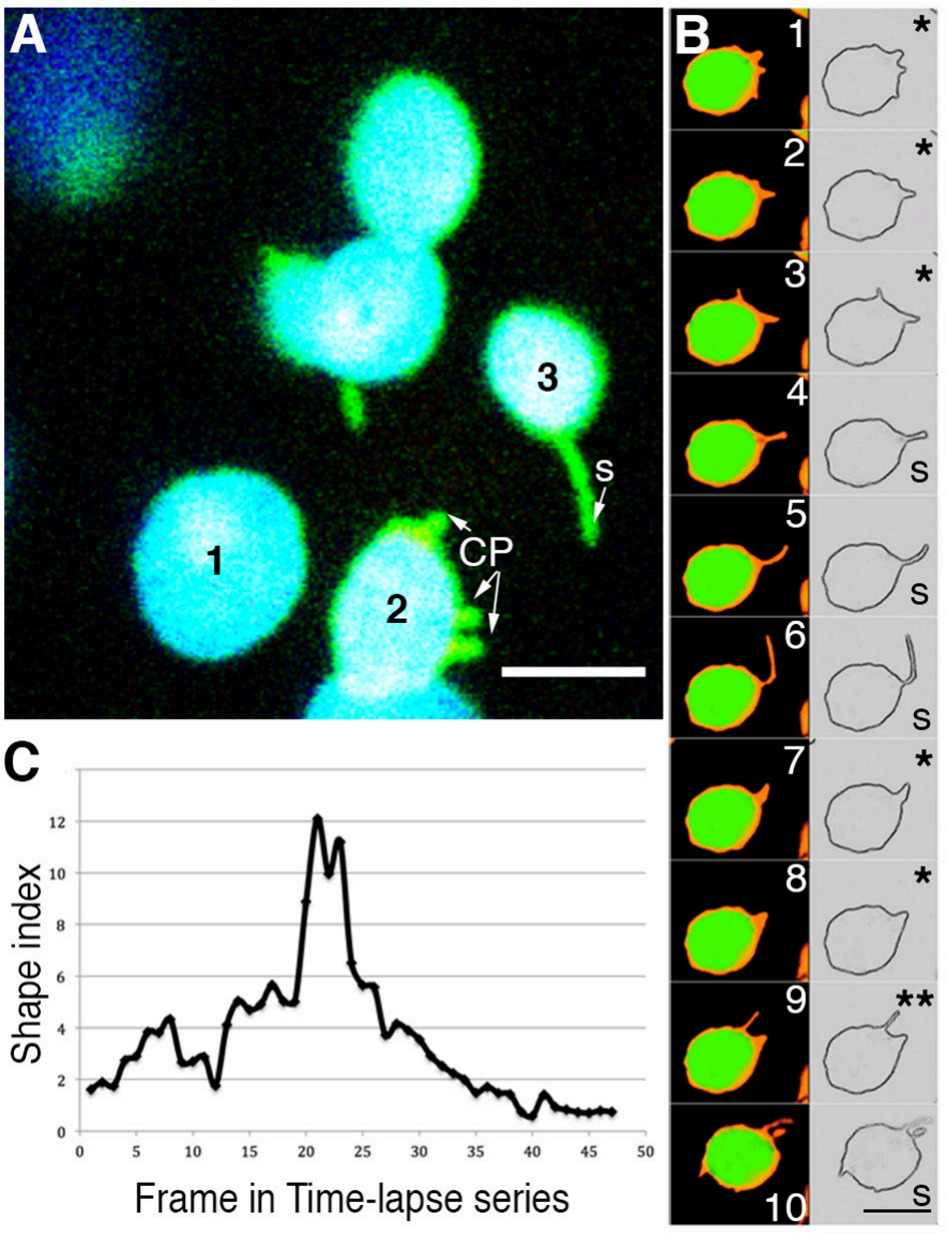
**Analysis of time-lapse image series of chloroplasts suggests that the terms chloroplast protrusions (CP) and stromules merely represent varying degrees of plastid extension**. **(A)** A snapshot pointing to three chloroplasts (chlorophyll depicted in blue; stroma-targeted GFP-green) in a single cell where plastid 1-does not exhibit any extension; based on a shape index (Holzinger et al., 2007b) plastid 2-exhibits small protrusions that are labeled CP; plastid 3-exhibits a clear tubular stromule (s). **(B)** Ten sequential images and their skeletonized version to show the plastid boundary have been taken from a time-lapse series of a single chloroplast from a plant expressing tpFNR:GFP (Movie 1). Depending upon which frame is being looked at the different stroma-filled (false colored orange) extensions and the plastid profile might be interpreted either as showing a CP (e.g., panels 1, 2, 3, 7, 8 marked with ^*^) or a stromule (panels 4, 5, 6, 10 marked with S). Panel 9 (^**^) shows two projections, the longer one suggesting a stromule while the shorter suggests a CP. Chlorophyll auto-fluorescence is false colored green. Size bar = 5 μm. **(C)** Graphic depiction of the continuously changing shape index of a single extension from a chloroplast. The extension was measured in each frame of a time-lapse video (Movie 1) as the ratio of the stromule length to it's radius at the base. Using static snapshots Holzinger et al. (2007b) had demonstrated that the average shape indexes may be grouped into two populations, one that averaged 0.8 ± 0.3 and the other at 7 ± 1.3. As analyzed here for a time-lapse series, over time a single extension can grown and shrink to span both of these categories.

The publications on CP have largely been based on TEM snapshots while the FP-aided observations on stromules elegantly reveal the dynamic nature of the plastid. Nevertheless, the distinction appears quite artificial and a report of chloroplast extensions in bundle sheath cells in rice leaves used the terms CP and stromules interchangeably after realizing that the plastid extensions observed might be placed into either category (Sage and Sage, 2009). In addition the excellent transmission electron micrographs of plastids in *Arisarum proboscideum* (Bonzi and Fabbri, 1975) depicted protrusions that today might just as easily be labeled stromules. On the other hand reports published well after the term stromule was introduced (Köhler and Hanson, 2000) persisted in presenting narrow tubules as CP (Figures 2E, 4A in Holzinger et al., 2007a; Figures 5.2D,F, 5.4C,E in Lütz et al., 2012).

As part of our critical appraisal we investigated the behavior of numerous plastids expressing stroma-targeted tp-FNR:GFP. We found that in a snapshot of any leaf expressing stroma-targeted FP might suggest some chloroplasts to be exhibiting CP and others stromules (Figure 2A). Time-lapse images (Figures 2B,C) show that all stromules, irrespective of whether they are from chloroplasts or any other plastid type, develop from small protrusions that might stretch into tubules of varying lengths and thickness and retract to produce beaked plastids (Movie 1).

## The notion of protein exchange between independent plastids

While the use of stroma-targeted GFP allowed plastid stromules to be visualized in living plant cells another FP-based technique involving fluorescence recovery after photo-bleaching (FRAP) was presented alongside to suggest a very important finding (Köhler et al., 1997). The finding was that stromules could interconnect plastids and GFP could flow between them (Köhler et al., 1997). This conclusion was reached by carrying out FRAP on elongated leucoplasts from tobacco roots expressing stroma-targeted GFP. Although the interconnection of plastids was not observed it was assumed that it must have taken place and would have involved stromules. Köhler et al. (1997) were able to demonstrate flow of GFP within a single plastid compartment. Presentation of the FRAP-based view on leucoplasts in reviews and textbooks established a general idea that all plastids are able to connect and exchange proteins with each other (Hanson and Köhler, 2006; Hanson and Sattarzadeh, 2008, 2011). This view challenges the unitary nature of a plastid but the precise mechanism of plastid fusion implied in this idea has still not been elucidated.

**Figure 3:**
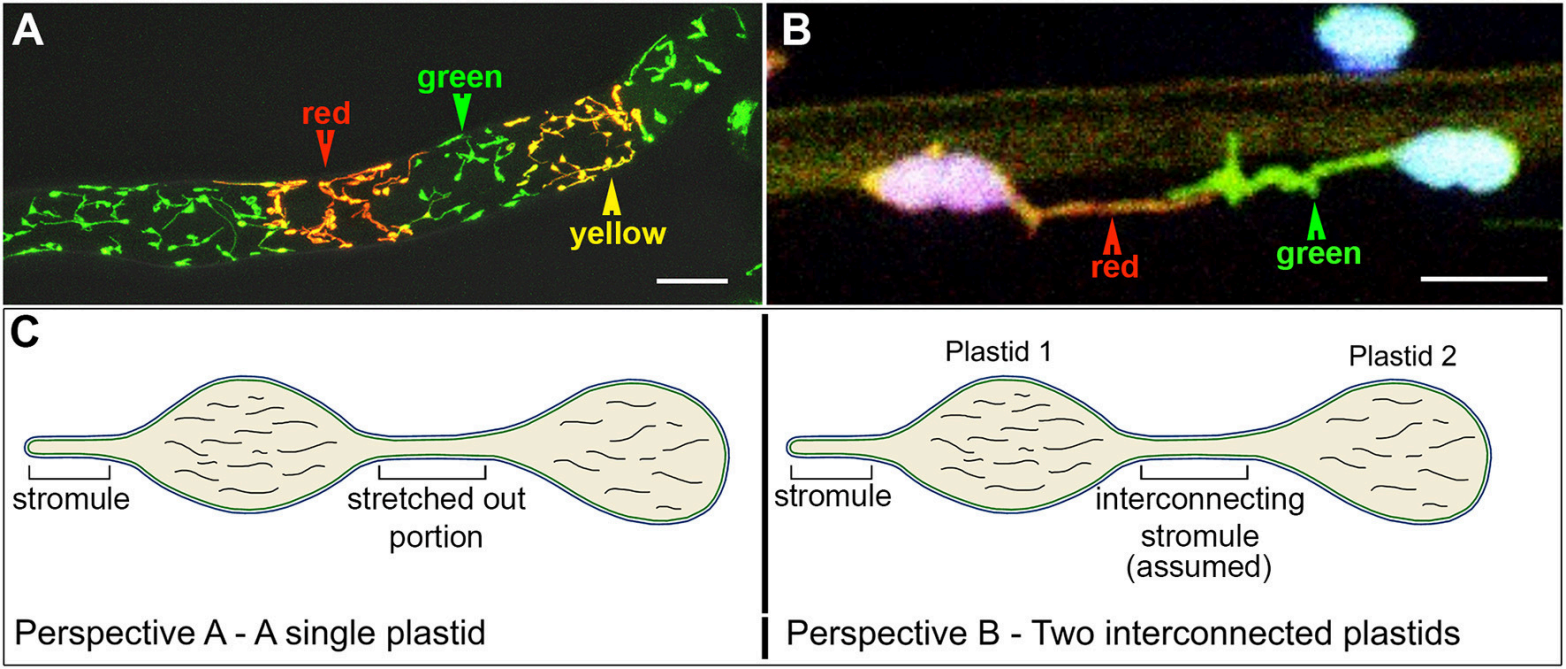
**The use of a stroma targeted green to red photo-convertible mEosFP for differential coloring of plastids allowed the long-standing idea of plastid-interconnectivity through stromules to be reassessed**. **(A)** A row of single cells showing leucoplasts in a tobacco BY2 cell line expressing the tpFNR:mEosFP shows the three colors (green, red, yellow) that are typically achieved using the probe. Non-photoconverted plastids and stromules appear green; after a 5–7 s exposure to 490 ± 30 nm light fully photoconverted leucoplasts appear red while yellow plastids are obtained after a short 2–5 s photoconversion period. **(B)** Chloroplasts in a pavement cell of a stably transformed Arabidopsis line expresing stroma-targeted tpFNR:mEosFP and chlorophyll (false colored blue) with extended stromules that appear to be interacting. Prolonged observations of hundreds of similar, differentially colored, dynamic plastids and stromules failed to show protein exchange between the chloroplasts. **(C)** Two perspectives of the plastid are presented. Perspective A interprets it as a single, elongated plastid with a narrow intervening tubular region such as that observed during normal pleomorphy of dynamic etioplasts, chromoplasts, and leucoplasts. This perspective is favored by Schattat et al. (2012a,b, 2015). Perspective B underlies the assumption of “interconnected plastids” and considers the narrow intervening region to be a stromule that connects two bulged domains considered as two independent plastid bodies. Leucoplasts with a very similar morphology were used in FRAP experiments to establish the idea of FP flow between plastids (Köhler et al., 1997). Whereas independent plastids actually becoming interconnected have not been observed the flow of a fluorescent protein from one point to another within a single, continuous, membrane bound compartment as depicted here can hardly be disputed. Size bar: **A** = 25 μm; **B** = 5 μm.

Meanwhile advances in FP technology resulted in the discovery and availability of monomeric Eos, a green to red photoconvertible fluorescent protein (Wiedenmann et al., 2004; Mathur et al., 2010) and allowed a stroma-targeted tpFNR:mEosFP probe to be created (Schattat et al., 2012a). This probe was originally designed to investigate the mechanism leading to protein exchange between plastids whose stromules exhibit prolonged interactions. The probe allows all plastids expressing it to be differentially colored in hues ranging from green to red (Figures 3A,B). Schattat et al. (2012a) reasoned that true fusion of stromules to inter-connect two independent plastids (e.g., Figure 3B) would result in a mixing of stromal color and provide an unequivocal demonstration of protein flow between two plastids. Alternatively maintenance of separate green and red plastid stroma colors despite apparent interaction between their stromules would suggest an inability to exchange fluorescent proteins. To demonstrate that the differential coloring technique and mixing of colors between two fusing organelles actually works they used mitochondria, which like plastids are also double membrane envelope bound organelles. Observations by Schattat et al. (2012a,b) and Mathur et al. (2013) did not support plastid fusion at all and thus strongly contradicted the FRAP-based work on root leucoplasts reported by Köhler et al. (1997). Despite the evidence that the plastid unit is maintained and no inter-plastid exchange of proteins is observed (Schattat et al., 2012a,b) Hanson and Sattarzadeh (2013) continue to support the original leucoplast-based findings of Köhler et al. (1997). The matter is therefore presently considered as a controversy.

An additional viewpoint propagated through literature based on stroma-targeted FPs suggested the occurrence of interconnected plastids (Köhler et al., 1997; Hanson and Sattarzadeh, 2013). This idea has also been challenged (Schattat et al., 2015), and it is noteworthy that with the exception of artificially initiated chloroplast fusion and in observations of senescent or diseased plant tissue, no one has actually observed two normal and independent plastid units fuse with each other. Further, etiolated plants often display etioplasts with two or more bulged regions connected by a thin tubule (Gunning, 1965; Schattat et al., 2015). Following exposure to light these regions, that appear very similar to plastid bodies, exhibit fluorescence as the protochlorophyllide changes into chlorophyll. As part of our critical appraisal the two views of an elongated plastid are summarized in Figure 3C.

## Insights from FPs targeted to plastid membranes

A number of probes localize to the three types of plastid membranes; the internal, thylakoid membranes; and the inner and outer membranes of the envelope (Table 1; Breuers et al., 2011). Many of the membrane-targeted probes have been expressed transiently under the constitutively active Cauliflower Mosaic Virus 35S promoter with a view of confirming their subcellular localization and are supported by biochemical evidence (Seo et al., 2009; Tan et al., 2011; Mueller et al., 2014). In several cases the overexpression of such fusion proteins has resulted in observations of protein patches on the plastid envelope (Seo et al., 2009; Tan et al., 2011). Alternatively it has led to the ectopic proliferation of membranes (Oikawa et al., 2008; Breuers et al., 2012). Specific patterns of extra membrane formation observed upon transient overexpression show that when proteins of the inner membrane such as AtTIC40:GFP are over-expressed multiple membrane layers are formed on the interior of the plastid envelope while outer membrane proteins such as AtTOC64:GFP form ectopic membrane extensions into the cytoplasm (Breuers et al., 2012; also see **Figure 5** for protein over-expression induced artifacts). Using electron microscopy the authors found that ectopic outer membrane formation was accompanied by a proliferation of the inner membrane and thus concluded that the membrane protrusions represented stromules. However, an electron microscopy based investigation generally does not provide as many chances of observing a phenomenon as provided by fluorescence microscopy of living cells. Thus, at present it is unclear whether all the protrusions formed due to overexpression of an outer membrane protein are actually stromules. Nevertheless, the observations of Breuers et al. (2012) provide an important and testable idea that membrane envelope remodeling such as that suggested during stromule formation might occur through changes in the protein: lipid ratio.

## FPs targeted to starch grains, plastoglobuli, and nucleoids

Two distinct types of storage products: starch and plastoglobuli are found in plastids. Starch is composed of long, branched polymers of glucose molecules and either takes a long term storage form, typically found in specialized leucoplasts called amyloplasts, or can transiently accumulate in photosynthesizing chloroplasts and be degraded subsequently during the dark period (Zeeman et al., 2010). Although several probes that target starch grains have been developed (Table 1; Figures 1C,D) and significant advances have been made in targeting FPs into economically important cereal (Primavesi et al., 2008; Wu et al., 2013; Krishnakumar et al., 2015) and tuberous crops (Sidorov et al., 1999;) their use in understanding the dynamic process of starch grain development is still rather limited. Similarly while biochemical and molecular analysis has identified mutants with different starch composition and properties the effect of different mutations on starch-accumulating plastids is just beginning to be assessed (Matsushima et al., 2014; Sun et al., 2014; Hara et al., 2015; Zhang et al., 2015). It is also notable that although many FP probes highlight plastids in roots the diurnal behavior of leucoplasts, their rapid response to stimuli such as gravity, physical barriers, water and nutrient stress and to soil microorganisms remain relatively unexplored areas of FP-based research.

In this context one particular starch probe that has remained underexploited is GBSS-GFP (Bahaji et al., 2011; Figure 1C). This probe exhibits dual localization; it highlights large starch grains, but when the grains are small or non-existent GBSS:GFP expressed under a CaMV35S promoter predominantly localizes to the stroma. This localization masks small starch grains in some plastid types and makes it a challenging probe for studying the early steps of starch formation. Since GBSS is found exclusively bound to the starch grain when chloroplast fractions are studied (Smith et al., 2004) the stromal localization might result from the 35S promoter induced overexpression or from altered fusion protein turnover due to the presence of GFP.

In contrast to starch plastoglobuli are found in nearly all plastids and their biochemical composition varies between plastid types. They can be formed from a wide variety of molecules including plastoquinone-9, plastoquinol-9, α-tocopherol, galactolipids, tri-acylglycerols, and carotenoids (Lichtenthaler, 2013). Since plastoglobules can be readily purified biochemically and are being subjected to proteomics (Ytterberg et al., 2006; Nacir and Bréhélin, 2013) the FP-probes for plastoglobuli (Table 1) are presently rather under utilized. However, the formation of plastoglobules, their spatio-temporal relation to thylakoids, their characteristic accumulation in different plastid types during development and their fuction in senescent tissues are all interesting questions that are beginning to be explored using live-imaging approaches (Nacir and Bréhélin, 2013; Shanmugabalaji et al., 2013). A very similar situation exists for plastid nucleoids that have been visualized (Figure 1E) but whose localization details during plastid development, differentiation and division await further exploration.

## FP-aided insights on plastid interactions

The endosymbiont theory for the origin of plastids also points to their interactions with all other components and compartments of the plant cell (Margulis, 1970). Plastid interactions have been suggested through organelle/membrane proximity in electron micrographs and concluded from biochemical investigations that have tracked plastid products such as sugars and lipids (Block and Jouhet, 2015; Kölling et al., 2015) as well as signaling components (Sandalio and Foyer, 2015 and cited publications) to other cytoplasmic structures. Several proteins exhibit dual or multiple localization patterns (e.g., Table 2), and whereas some of the localizations in transient expression studies might turn out to be artifacts others suggest biochemical relationships shared between different organelles. Some localization patterns might reflect a condition specific status. In addition recent years have seen widespread availability of various FP-probes for plastids and other organelles (Mathur, 2007; The Illuminated Plant Cell, <http://www.illuminatedcell.com>; Mano et al., 2011; The maize GFP data base <http://maize.jcvi.org/cellgenomics/index.php>) and these have been very useful in establishing views regarding plastid interactions with other organelles. Some of the resultant insights are presented.

**Table 2:** **Some proteins that show multiple localizations**.

**Localization**		**Organism/cell type**	**Protein**	**FP**	**Key features**	**References**
Dual	Chl ER	*C. reinhardtii*	RB60	G	Protein disulfide isomerase; part of redox regulatory protein complex involved in translation in chloroplasts; exists as soluble form in stroma or tightly bound to thylakoid membrane; also retained in the ER	Levitan et al., 2005
	Chl ER	*N. benthamiana*	BnCLIP1	G	Lipase; MCS between plastids and ER. Putative plastid inner membrane of envelope localized	Tan et al., 2011
	Chl P	*A. thaliana*	DRP5B (ARC5)	G	Chloroplast and peroxisome fission; cytosolic, recruited to a discontinuous ring around membrane fission sites	Zhang and Hu, 2010
	Chl Cyt	*P. patens*	FtsZ	G	Part of division ring; cytosolic assembles into a ring in chloroplasts	Kiessling et al., 2004
	Chl M	*A. thaliana*	*At*DEF1	G	Peptide deformylase; catalyzes *N*-formyl group removal from methionine residues of nascent polypeptides; AtDEF1.2 and AtDEF2 found in stroma and thylakoid; AtDEF1.1 localizes to mitochondria	Dinkins et al., 2003
	Chl M	*A. thaliana*	MST1	G	Mercaptopyruvate sulfurtransferase	Nakamura et al., 2000
	Chl M	*N. tabacum*	AtHRS1	G	Histidyl-tRNA synthetase	Akashi et al., 1998
	Chl M	*O. sativa*	Virescent2 (V2)	G	Plastid and mitochondrial guanylate kinase (pt/mtGK)	Sugimoto et al., 2007
	Chl M	*Z. mays*	ZmSig2B	G	Nucleus-encoded sigma factor; accumulates in chloroplasts and mitochondria	Beardslee et al., 2002
	Pl M	*G. max*	glutathione reductase	G	Component of ascorbate-glutathione cycle	Chew et al., 2003
	Pl M	*Z. mays*	Myosin XI	Ab^*^	Myosin motor protein	Wang and Pesacreta, 2004
	Pl	Oryza spp.	OsNIN1 (M)	G	Alkaline/neutral invertase; transported into both mitochondria and plastids	Murayama and Handa, 2007
	M		OsNIN3 (Pl)	G		
	Pl	*A. thaliana*	AtGLR3.4	Y	Glutamate receptor	Teardo et al., 2011
	Pm	*N. tabacum*				
	Pl Vac	*A. thaliana*	ATG8	G	ATG-dependent autophagy; co-localizes with stroma-targeted DsRed in RCBs in vacuoles	Ishida et al., 2008
Triple	Chl Cyt M	*A. thaliaa*	tRNA nucleotidyl transferase	G	Adds 3'-terminal cytidine–cytidine–adenosine to tRNAs	von Braun et al., 2007
	Chl/Pl	*A. thaliana*	FIS1A	Y	Tail anchored membrane protein; implicated in mitochondrial and peroxisomal fission	YFP: Ruberti et al., 2014
	P			E		mEosFP: Jaipargas, 2015
	M					
	Chl/Pl ER- Go	*A. cea*	Amyl-1	G	α-amylase isoform; localized in amyloplasts degrades starch	Kitajima et al., 2009

Chl, chloroplasts; Pl, plastids; Pm, plasma membrane; P, peroxisomes; M, mitochondria; ER, endoplasmic reticulum; N, nucleus; Go, Golgi bodies; Vac, vacuole; Ly, Lysosomes; E, mEosFP; G, GFP; Y, YFP; ^*^Ab, antibodies were used, not FP.

## Plastids and the cytoskeleton

Plants need light in order undergo photosynthesis. Photosynthesis takes place in the chloroplasts of plants but too much or too little light can have negative effects on plant health. Plants have developed two chloroplast responses to combat the lack of or excess of light, the chloroplast accumulation and avoidance responses (Sakai et al., 2001; Kagawa et al., 2004; Wada, 2013). Chloroplasts have been shown to accumulate on the irradiated side of the cell under low intensity blue light, or move away from the light source under high light intensity (Sakai et al., 2001; Kagawa et al., 2004). Two photoreceptors phototropin 1 and phototropin 2 (PHOT1, PHOT2) are implicated in mediating this response (Briggs et al., 2001; Sakai et al., 2001). The light avoidance response possibly minimizes chloroplast damage, thus saving photosystem II (Kasahara et al., 2002, 2004; Takahashi and Badger, 2011) and is mediated by F-actin that surrounds a chloroplast (cp-actin; Kandasamy and Meagher, 1999; Kadota et al., 2009). The cp-actin appears to facilitate chloroplast movement in both the accumulation and avoidance responses through the formation and disassociation of cp-actin on the leading edge and the trailing end of the chloroplast, respectively (Kadota et al., 2009). Major insights have come from analyses of the CHLOROPLAST UNUSUAL POSITIONING gene (CHUP1) and different FP-fusions of its domains and the chup1 mutant (Oikawa et al., 2003; Schmidt von Braun and Schleiff, 2008; Lehmann et al., 2011). The involvement of myosin motor proteins in plastid movement has been strongly indicated (Paves and Truve, 2007; Kong and Wada, 2011; Wada, 2013).

The involvement of cytoskeletal elements and motor proteins in stromule extension was also investigated (Kwok and Hanson, 2003). The use of different cytoskeleton inhibitors suggested that the formation of stromules and their behavior relies to different degrees upon both microfilaments and microtubules (Kwok and Hanson, 2003). The myosin ATPase inhibitor 2,3-butanedione 2-monoxime (BDM) also resulted in decresed stromule dynamics and suggested the involvement of myosin motors (Gray et al., 2001). Subsequently using transient RNA interference of myosin XI and by localizing a GFP fused to the tail domain of this motor protein to the chloroplast envelope, again in transient expression Natesan et al. (2009) concluded that myosins are essential for stromule formation. Notably, their transient expression based observations using the cargo domain of myosin XI fused to GFP suggest a rather non-specific localization as it includes several other organelles (Natesan et al., 2009). Another transient expression based study using a trucncated version of myosin XI reached a similar conclusion (Sattarzadeh et al., 2009).

## Plastids and the endoplasmic reticulum

**Figure 4:**
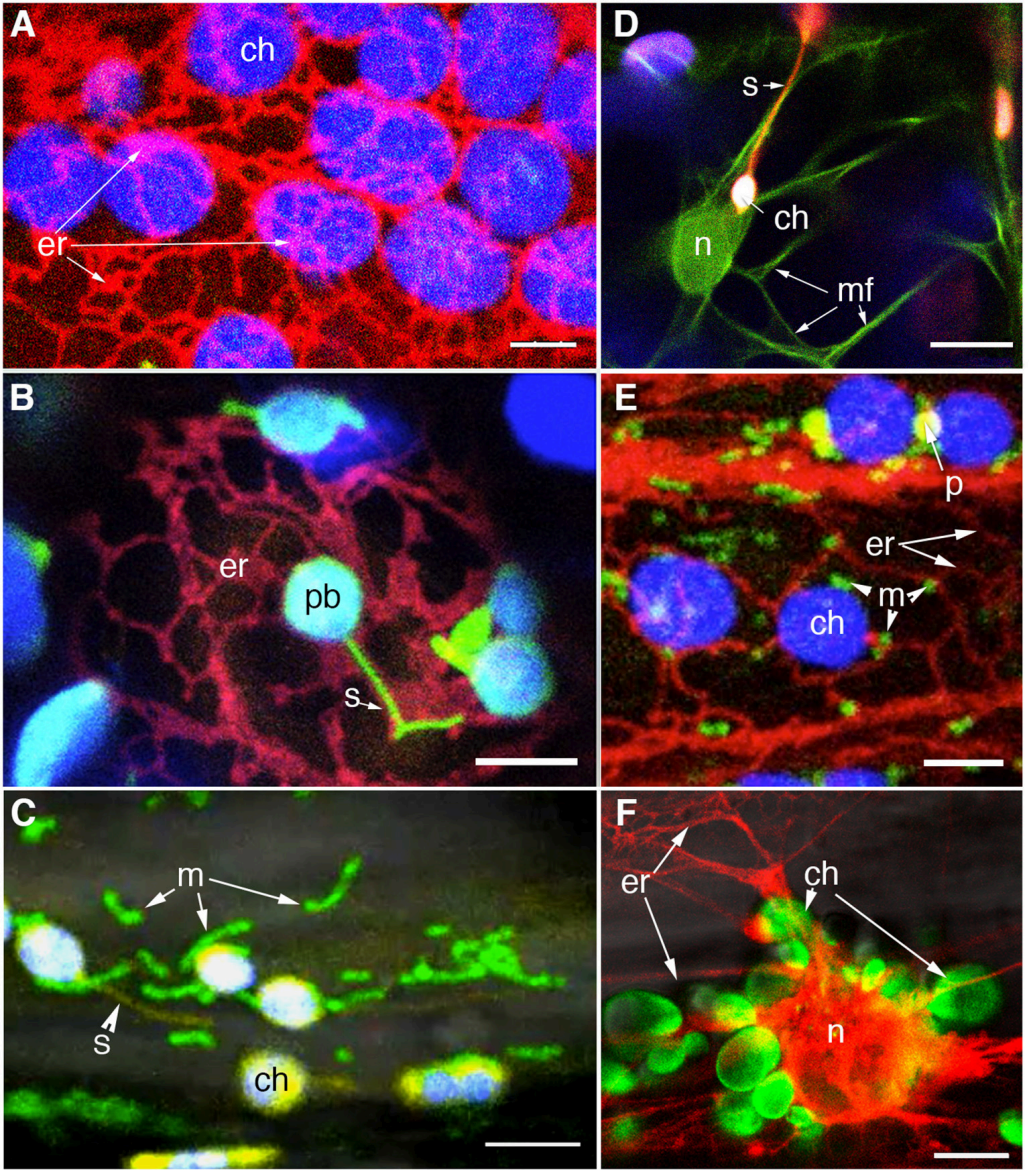
**Visualization of different colored FP to specific organelles facilitates investigations on plastid interactions**. **(A)** Confocal image of chloroplasts (chlorophyll autofluorescence false colored blue) and RFP-highlighted ER shows the ER-cage around plastids in a stable transgenic Arabidopsis line. **(B)** An Arabidopsis line co-expressing stroma-targeted tpFNR:GFP [green; plastid body (pb) with chlorophyll false colored blue] and RFP targeted to the ER allowed the stromule (s) -ER correlation to be investigated (Schattat et al., 2011a,b). **(C)** A stable transgenic line coexpressing stroma targeted tpFNR:YFP and mito:GFP (Logan and Leaver, 2000) is allowing an investigation on the mitochondria (m) relationship to chloroplasts (ch) and stromules (s). **(D)** Investigations on F-actin (mf) relationship to chloroplasts (ch) and stromules (s) are being facilitated through a double transgenic line expressing GFP:mTalin (Kost et al., 1998; green) and tpFNR:mEosFP (red). F-actin around the nucleus (n) is apparent. **(E)** A small region from a hypocotyl cell of a triple transgenic expressing RFP targeted to the ER (er), GFP targeted to mitochondria (m) and a YFP targeted to peroxisomes (p). Chloroplasts (ch) are discernable due to their autofluorescence. The line is being used for investigating the relationship between the four organelles. **(F)** A double transgenic line co-expressing tpFNR:GFP and RFP-ER shows the peri-nuclear ER cage and the cluster of chloroplasts (ch) surrounding the nucleus (n) in a hypocotyl cell from a dark grown seedling. The probes might provide several interesting observations and insights into retrograde signaling between plastids and the nucleus. Size bars: **A–C,E,F** = 5 μm; **D** = 10 μm.

Electron microscopy based investigations have indicated intimate connections between the plastid and the endoplasmic reticulum (ER) membranes (Wooding and Northcot, 1965; McLean et al., 1988; Whatley et al., 1991). However, a clear demonstration of plastid and ER interactivity was achieved through simultaneous imaging of different colored FPs targeted to the two organelles (Schattat et al., 2011a,b; Figure 4). A loose ER cage around the plastid body (Figure 4A), and stromules co-aligned with ER tubules (Figure 4B) were observed. The organellle interactivity suggested by these observations was attributed to the presence of membrane contact sites (MCS) between the plastid envelope and the ER (Schattat et al., 2011a,b). The presence of MCS and their strong interconnectivity has been suggested through laser optical tweezers assisted pulling of GFP-labeled ER strands attached to chloroplasts (Andersson et al., 2007). In addition a chloroplast localized lipase from *Brassica napus* fused to GFP (BnCLIP1:GFP) that shows co-localization with ER tubules has been interpreted as indicative of MCS (Tan et al., 2011). While the precise nature of plastid-ER interactions remains to be characterized the identification of the trigalactosyldiacylglycerol (TGD) transporter complex and its association with the ER during lipid biosynthesis are promising leads that are being actively pursued (Xu et al., 2008, 2010; Block and Jouhet, 2015).

## The plastid-nucleus relationship and views on retrograde signaling during response to pathogens

As purported descendants of prokaryotic endosymbionts and possessing their own genetic and protein machinery chloroplast gene expression must be highly coordinated with nuclear encoded genes in order to maintain optimal functionality within the cell. Indeed observations of plastids clustered around the nucleus in different epidermal cells with stromules ramifying the grooves and infoldings of the nuclear envelope (Kwok and Hanson, 2004b; Figure 4F) favor the idea of signaling between the two organelles. Retrograde signaling from chloroplasts to the nucleus is known to depend upon exposure to light and the redox state of the plastid, might be mediated through metabolite sensing as well as reactive oxygen species (ROS), and involve plastid membrane bound transcription factors (Fernández and Strand, 2008; Stael et al., 2014; Chi et al., 2015). Fluorescent proteins have proved useful in understanding this aspect of plastid integration within the cell.

An elegant approach to understand retrograde signaling from the plastid during pathogen response was taken to follow the movement of N-Receptor Interacting Protein 1 (NRIP1) from chloroplasts to nuclei using NRIP1 fused to the Cerulean fluorescent protein with an N-terminal nuclear export signal (NES) (Caplan et al., 2015). NES-NRIP1-Cerulean can only accumulate within the nucleus after it has been imported and processed within the chloroplast, where the chloroplast transit peptide of NRIP1 is cleaved off along with the NES. Movement of NRIP1, which accumulates within the chloroplast, to the nucleus is triggered in response to Tobacco Mosaic Virus (TMV) infection or expression of the TMV effector protein p50. When NES-NRIP1-Cerulean was co-expressed with p50, processed NES-NRIP1-Cerulean accumulated within the nucleus while no such accumulation was observed when NES-NRIP1-Cerulean was expressed alone. The observation that during the response to p50 expression stromules and plastid bodies can be found in close proximity to the nucleus has been used to suggest that stromules are involved in the direct movement of NRIP1 to the nucleus during the innate plant immune response (Caplan et al., 2008, 2015).

Stromules have also been implicated in facilitating plastid-to-nucleus trafficking during infection of *N. benthamiana* with *Abutilon* mosaic virus (AbMV; Krenz et al., 2010, 2012). Using BiFC (Bimolecular Fluorescence Complementation) as interaction between the AbMV movement protein (MP) and the plastid localized heat shock cognate 70 kDa protein (cpHSC70-1) was observed regardless of infection; however, when challenged with AbMV infection the number of plastids producing stromules as well as the length of the stromules was increased (Krenz et al., 2010, 2012). Similar observations made using the outer envelope protein-7 (OEP7) during AbMV infection have led to the proposal that stromules are involved in the trafficking of AbMV MP from the cell periphery to the nucleus, or vice-versa, during the infection process (Krenz et al., 2010, 2012, 2014).

These observations are interesting and the conclusions derived from them seem very well thought out. However, it is difficult to reconcile the direct involvement of stromules in the retrograde signaling since none of the studies appear to consider the diurnal fluctuations that lead to stromule extension and retraction. The diurnal cycle of stromules either as a response to a change in chloroplast redox status or a change in cellular sugar levels is quite clear (Schattat and Klösgen, 2011; Schattat et al., 2012a; Brunkard et al., 2015). What happens to the postulated retrograde signaling at night when stromules are not extended? Perhaps the observations are the result of a physiological perturbation of the cell during infection and not indicative of a function of stromules (Krenz et al., 2012). Indeed previous work has interpreted Geminivirus-induced plastid alterations to perturbed carbon metabolism that is likely caused by the disruption of sugar translocation through phloem during infection (Jeske and Werz, 1978). The use of *Agrobacterium* mediated overexpression of proteins under consideration again suggests caution in the interpretations since *Agrobacterium* infiltration itself has been shown to increase stromule frequency (Schattat et al., 2012b; Erickson et al., 2014). Furthermore, as the development of AbMV is known to be affected by light intensity as well as diurnal and seasonal conditions (Krenz et al., 2012), observations linking AbMV infection and stromule formation should be reconsidered to account for the diurnal rhythm of stromule formation (Schattat et al., 2012a; Brunkard et al., 2015) and how the plant's response to a pathogen might affect this cycle. Similarly, given the importance of a plant's developmental stage in relation to stromule formation (Waters et al., 2004) it would interesting to extend these observations over the course of development in both challenged and unchallenged plants instead of assessing a single time point following infection. Although Caplan et al. (2015) conclude that stromules are involved in the direct transfer of processed NES-NRIP1-Cerulean to the nucleus, it is equally possible that after cleavage of the NES signal NRIP1-Cerulean leaks to the cytosol and then accumulates in the nucleus. Accumulation of an untargeted FP in the nucleus is one of the major caveats associated with their use (Haseloff et al., 1997; Mathur et al., 2010). Interestingly many of the observations involving pathogens span several days without really describing or characterizing the state of the cells or the plastids during those days. Furthermore, clustering of plastids and stromules around the nucleus is not restricted to pathogen response and can be observed throughout the normal development of plants (Kwok and Hanson, 2004b; Figure 4F).

We conclude that the coincidental observations of stromules in virus or other pathogen infected tissue and the suggestion that stromules facilitate retrograde signaling between the plastid and nucleus is a possibility but at present it does not fit in into the well-documented diurnal phenomenon of stromule extension and retraction.

## Targeted FPs have provided a comprehensive view of the plastid division process

In higher plants plastid division by binary fission involves a coordinated assembly of four concentric division rings that together constrict both the inner and outer membranes of the plastid envelope (Osteryoung and Pyke, 2014). Whereas some of the proteins such as the internal ring localized FtsZ appear to be of prokaryotic origins others such as the ARC5/DRP5B indicate a eukaryotic derivation. Fluorescent proteins have been used to confirm the localization of several division related proteins at the mid-plastid division site as well as provide convincing proof for their sequential activity through complementation of the pertinent mutant (Vitha et al., 2001; Gao et al., 2003; Miyagishima et al., 2006; Fujiwara et al., 2008; Glynn et al., 2008, 2009; Nobusawa and Umeda, 2012). Using FP-probes it was determined that FtsZ proteins are the first to align on the mid-plastid (Vitha et al., 2001). In subsequent experiments the expression of ARC5-GFP in *pdv1 pdv2* mutants showed impaired localization of ARC5 and led to the conclusion that PDV proteins are necessary for ARC5 localization (Miyagishima et al., 2006). Glynn et al. (2008) performed similar experiments to determine that ARC6 is required to recruit PDV2 to the division ring. FP-based observations have thus provided a comprehensive understanding of the construction of the plastid division ring (Nakanishi et al., 2009; Osteryoung and Pyke, 2014). Additional information on the phenomenon was obtained by using a GFP fused to a bacteria-derived FtsZ1 to assess the effects of higher or lower FtsZ1 expression on division efficiency (Vitha et al., 2001). In other experiments, the use of FtsZ2-GFP probes to observe division ring formation in the presence or absence of cafenstrole, an inhibitor of very-long-chain fatty acids (VLCFA) synthesis, provided an insight on the involvement of VLCFAs in plastid division (Nobusawa and Umeda, 2012).

## Insights into plastid breakdown using FPs

Senescence is an integral part of the plant's life cycle and involves orchestration of physiological changes designed to recapture and recycle cellular resources. Chloroplasts are amongst the more robust cellular elements and in many tissues are the last to disappear. Senescent chloroplasts, also called gerontoplasts (Figure 1E), appear swollen and often display an amoeboid behavior. They also acquire very different behavioral and biochemical characteristics as compared to healthy chloroplasts (Wise, 2007). At the ultra-structural level gerontoplasts exhibit a progressive un-stacking of grana, a loss of thylakoid membranes and a massive increase in the number of plastoglobuli (Harris and Arnott, 1973; Krupinska, 2007). The controlled disassembly of the photosynthetic apparatus often resembles autophagy (Ishida et al., 2014; Izumi et al., 2015) and results in the formation of vesicles containing stromal and thylakoid material (Krupinska, 2007; Figure 1E). Amongst the degradation-vesicles are the Rubisco-containing bodies (Chiba et al., 2003) that have been observed using stroma-targeted FPs (Ishida et al., 2008; Yamane et al., 2012).

## FPs are useful in learning about plastid associated reactive oxygen species

Reactive oxygen species (ROS), such as hydrogen peroxide (H_2_O_2_), are commonly thought of as toxic molecules leading to cellular damage; primarily through lipid peroxidation and membrane degradation. There is an obvious association between the increase of different ROS within the cell during senescence as well as during abiotic and biotic stresses (Zentgraf, 2007; Foyer and Noctor, 2009). Several recent studies have employed fluorescent proteins, such as the redox sensitive GFP (roGFP) (Jiang et al., 2006; Meyer et al., 2007; Schwarzländer et al., 2008) to measure subcellular redox states within living plant cells. The roGFP is sensitive to reduced glutathione pools within the cell which, with the help of endogenous glutaredoxin, reduces roGFP and produces a disulfide bridge between two cysteines that have been engineered into roGFP (Sugiura et al., 2015). Formation of the disulfide bridge causes a conformational change that shifts the excitation maxima and allows ratiometric quantitation of the reduced glutathione pool within a living cell (Hanson et al., 2004; Jiang et al., 2006; Meyer et al., 2007; Schwarzländer et al., 2008; Sugiura et al., 2015). Another FP used to directly estimate the relative concentrations of H_2_O_2_ is the modified YFP known as HyPer (Costa et al., 2010). This probe comprises of YFP fused to a regulatory domain of the *Escherichia coli* H_2_O_2_ sensor OxyR. When HyPer is exposed to H_2_O_2_, two cysteine bonds form within the OxyR and produce a conformation-induced shift in the excitation maxima from 420 to 500 nm, while the emission maximum of 516 nm remains constant, to allow a ratiometric measurement of H_2_O_2_ (Belousov et al., 2006).

HyPer was first characterized in plants using the guard cells of stable transgenic *Arabidopsis* as well as in suspension cell cultures obtained from these plants where a dosage dependent increase in cytosolic HyPer fluorescence was observed following treatments with exogenous H_2_O_2_ (Costa et al., 2010). HyPer has since been used to assess the response of plastids to H_2_O_2_ produced during pathogen response and to investigate potential plastid-to-nucleus signaling via plastid produced H_2_O_2_ (Caplan et al., 2015). Using a chloroplast targeted HyPer, Caplan et al. (2015), demonstrated that following expression of p50 in *N. benthamiana*, which is known to elicit ROS bursts and augment H_2_O_2_ levels the stromule frequency also increased. Furthermore, when chloroplasts clustered closely around a nucleus were scanned with a 405 nm laser to generate light-induced ROS in chloroplasts, the fluorescence intensity of nuclear localized NLS-HyPer increased; indicating that chloroplast generated H_2_O_2_ accumulated in the nucleus and could be involved in chloroplast to nucleus signaling (Caplan et al., 2015). These studies clearly demonstrated the utility of HyPer in assessing H_2_O_2_ levels within different compartments of the plant cell. It will be interesting to see whether these probes can be applied to investigate changes in cellular redox states during other stresses.

In addition to senescence associated plastid degradation the breakdown of chloroplasts is also linked to a programmed cell death phenomenon that occurs under oxidative stress produced by exposure to high light or physical injury (Apel and Hirt, 2004). The plastid-associated PCD involves the release of singlet oxygen (^1^O_2_) and leads to the formation of micro-lesions without impairing the general viability of the plant. In green tissue one of the first signs of this localized phenomenon is the loss of chloroplast integrity. An elegant FP-based assay estimated the damage to chloroplasts by observing the leakage of stroma-targeted GFP into the cytoplasm following the ^1^O_2_ stress (Kim et al., 2012).

## Targeted FPs and identifying the potential for artifacts

**Figure 5:**
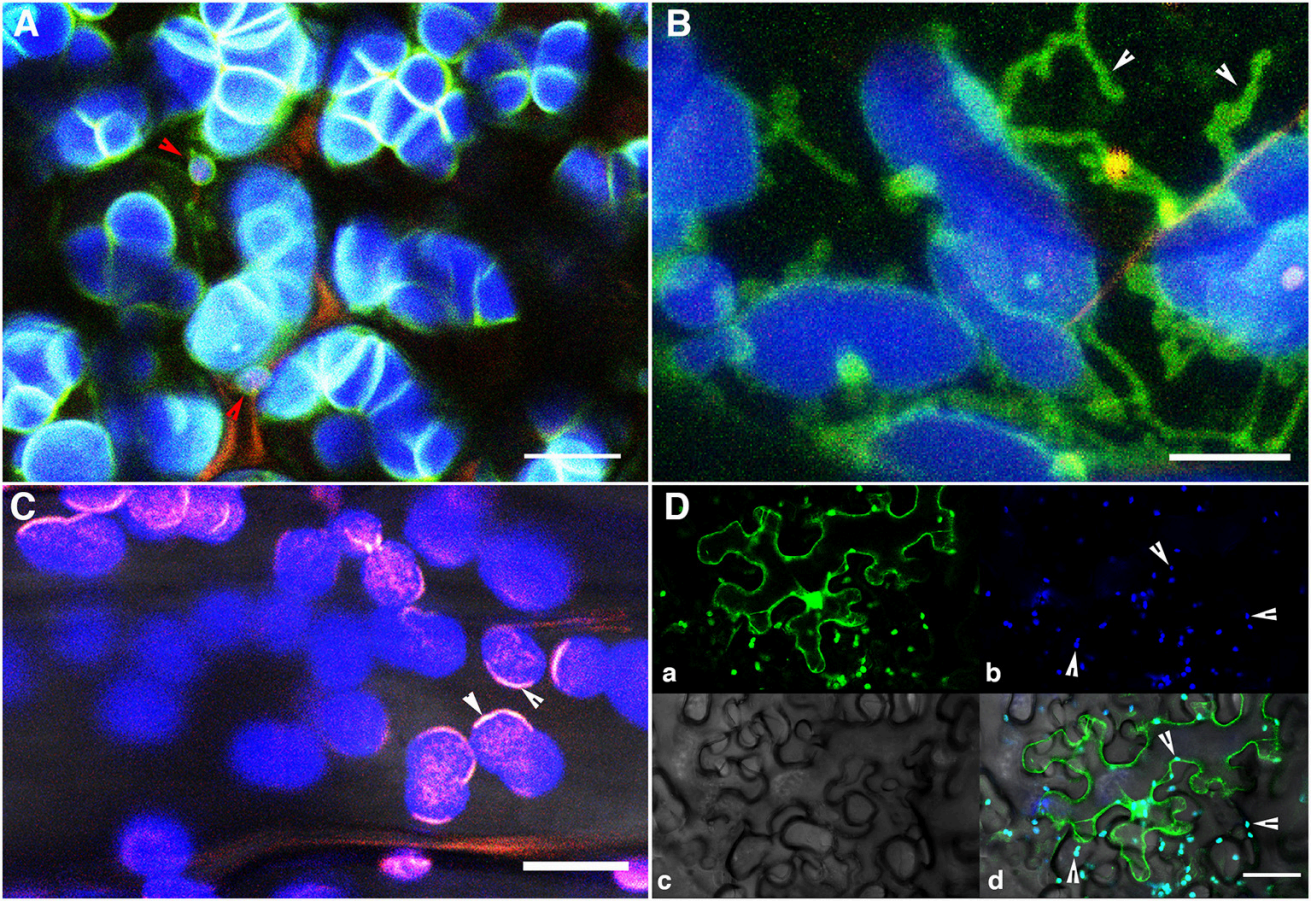
**Some of the artifacts resulting from overexpression of a fusion protein**. **(A)** Overexpression of a N-CHUP:GFP fusion results in sticky plastid envelopes and their massive clumping. **(B)** N-CHUP:GFP overexpression may also result in ectopic protrusions resembling stromules. Whether all such protrusions are actually stromules remains to be determined. **(C)** OE of FIB4:mEosFP that normally localizes to plastoglobuli (Figure 1D) can also produce localized artifacts such as extra lining of the inner membrane of the envelope. Observation made using transient expression in tobacco cells. **(D)** Leakage of stroma-targeted FP due to pressure/touch—induced damage to the cell makes the cytoplasm fluoresce due to mis-localization. Note the presence of chloroplasts in pavement cells. Size Bar = 5 μm in **(A,B)**; 10 μm in **(C)**; 50 μm in **(D)**.

As reviewed here the use of FPs has resulted in several commendable insights on plastids. However, it is important to remember that any fusion protein, despite its expression under the control of the cellular machinery in a living plant cell, is still an artificially created chimera that is quite different from the tag-free protein under investigation. In general, the addition of a 20–30 kDa FP changes the properties of a protein, including its stability and turnover characteristics. In addition the expression of many FP-fusions is augmented through the use of the strong CaMV-35S, or even a double 35S promoter, and thus does not represent the actual protein levels that would be achieved under the native promoter. FP-fusions, specifically those targeted to the plastid membranes are prone to zippering and clumping (Figure 5A), can produce abnormal aggregates and large patches, lead to ectopic protrusions (Figures 5B,C) and sometimes even provide wrong localizations due to overexpression. Whereas transient expression of fusion proteins is quite efficient and relatively easy to perform it results in a wide range of protein expression levels that vary with time. Such heterogeneity of gene expression promotes a “pick and choose” approach that may bias the observations and resultant conclusions. The creation of multiple stable transgenic lines expressing a specific construct allows for more convincing observations that can be revisited, be subjected to more critical assessments, be studied under different growth and development conditions, and most importantly, can be verified by other investigators. However, transgenic plant creation does require much more time and labor.

A common practice for most plant labs involves the transient agroinfiltration technique where fusions for Arabidopsis genes might be carried out in *N. benthamiana* or other tobacco species. While non-matching observations are not usually reported it is worth noting that transient expression patterns obtained using tobacco plants or single cell cultures are not always replicated in stable transgenic Arabidopsis plants. Again, consistency between materials chosen for agroinfiltration remains an important factor since young leaves are physiologically quite different from older, fully expanded leaves, which in turn are very different from senescent ones. For plastids specially, this is an important criterion as the plastid types between green tissue and yellow-green (senescent/stressed) tissue are different. A technique being used quite often involves virus induced gene silencing (VIGS). Studies aimed at understanding stromules must consider the developmental stage of the plant being used as it can have a major effect on the overall conclusions.

During our critical appraisal of published literature on stromules we have become aware of a major discrepancy. Whereas several researchers report and emphasize the diurnal nature of changes in plastid morphology involving the extension and retraction of stromules (Schattat et al., 2011a,b, 2012a; Brunkard et al., 2015) others completely overlook this important fact and draw conclusions from observations that stretch into several days and even weeks. At this stage we can only wonder if conclusions obtained after prolonged periods on a subcellular phenomenon that is observable within 2–5 h should still be worthy of consideration. A very similar reasoning requiring attention concerns inferences on ROS mediated effects on plastid behavior. The term ROS encompasses many different types of oxygen species, each with a different lifetime that affects its ability to penetrate, interact and alter the behavior of cellular membranes (Foyer and Noctor, 2009). Different ROS also trigger chain reactions that can involve several other ROS as well as reactive nitrogen species (RNS). The commonly available point-scanning laser microscopes are not usually calibrated to deal with the small time scales involved in ROS induced changes and provide real-time data. Whereas the emission of ROS as a general stress induced occurrence in living cells cannot be challenged the estimation of a single ROS through fluorescence decay of a specific FP cannot be indicative of the true ROS levels and the perturbations caused by them in a living cell.

## Future prospects for FP based investigations on plastids

Despite the considerable advances in knowledge about plastids where the use of FPs has played an important role some very important questions about these essential organelles of plant cells remain unanswered. The recognition that plastids are independent functional units still requires unequivocal proof. If this idea has to hold true then it should be possible through the use of FPs to distinguish between plastids that look similar but might be metabolically dissimilar. Further, much of our information on plastids comes from the study of chloroplasts. FPs targeted to other plastid types might allow us to fully comprehend the versatile and inter-convertible nature of these organelles. The availability of probes that are already targeted to plastid inclusions such as starch and plastoglobuli suggests that we could start now start combing these probes with an aim to investigate carbon partitioning within plastids. Although some investigations have been carried out on the interactions between plastids, mitochondria and peroxisomes (Kwok and Hanson, 2003, 2004b,c; Jouhet et al., 2004; Mathur et al., 2012) more details are expected to emerge from double and triple transgenic plants (Figure 4). It will be interesting to actually observe inter-organelle co-operation during photorespiration and high-stress conditions to perhaps add more information to that built up on from seminal TEM and biochemical studies.

## Author contributions

All authors provided input in writing this manuscript.

### Conflict of interest statement

The authors declare that the research was conducted in the absence of any commercial or financial relationships that could be construed as a potential conflict of interest.

## Supplementary material

Movie 1 **Time-lapse imaging of a single chloroplast to show that the difference between a chloroplast protrusion (CP) and a stromule is completely artificial since the two terms represent snapshots of a dynamic phenomenon**. Whereas this chloroplast is quite dynamic other plastids might not show pronounced extensions specially if the cytoplasm is also in a relatively low-dynamic mode. False colored chlorophyll autofluorescence is in green and the stroma in orange (See Figure 2).

## References

[B1] Abdel-Ghany S. E. Müller-Moulé P. Niyogi K. K. Pilon M. Shikanai T. (2005). Two P-type ATPases are required for copper delivery in *Arabidopsis thaliana* chloroplasts. Plant Cell 17, 1233–1251. 10.1105/tpc.104.030452 15772282 PMC1087999

[B2] Akashi K. Grandjean O. Small I. (1998). Potential dual targeting of an *Arabidopsis* archaebacterial-like histidyl-tRNA synthetase to mitochondria and chloroplasts. FEBS 431, 39–44. 10.1016/S0014-5793(98)00717-0 9684861

[B3] Albrecht V. Ingenfeld A. Apel K. (2006). Characterization of the *snowy cotyledon 1* mutant of *Arabidopsis thaliana*: the impact of chloroplast elongation factor G on chloroplast development and plant vitality. Plant Mol. Biol. 60, 507–518. 10.1007/s11103-005-4921-0 16525888

[B4] Andersson M. X. Goksor M. Sandelius A. S. (2007). Optical manipulation reveals strong attracting forces at membrane contact sites between endo- plasmic reticulum and chloroplasts. J. Biol. Chem. 282, 1170–1174. 10.1074/jbc.M608124200 17077082

[B5] Apel K. Hirt H. (2004). Reactive oxygen species: metabolism, oxidative stress, and signal transduction. Annu. Rev. Plant Biol. 55, 373–399. 10.1146/annurev.arplant.55.031903.141701 15377225

[B6] Arimura S. Hirai A. Tsutsumi N. (2001). Numerous and highly developed tubular projections from plastids observed in tobacco epidermal cells. Plant Sci. 160, 449–454. 10.1016/S0168-9452(00)00405-2 11166431

[B7] Armbruster U. Pribil M. Viola S. Xu W. Scharfenberg M. Hertle A. P.. (2013). *Arabidopsis* CURVATURE THYLAKOID1 proteins modify thylakoid architecture by inducing membrane curvature. Plant Cell 25, 2661–2678. 10.1105/tpc.113.113118 23839788 PMC3753390

[B8] Arsova B. Hoja U. Wimmelbacher M. Greiner E. Üstün Ş. Melzer M.. (2010). Plastidial thioredoxin z interacts with two fructokinase-like proteins in a thiol-dependent manner: evidence for an essential role in chloroplast development in Arabidopsis and *Nicotiana benthamiana*. Plant Cell 22, 1498–1515. 10.1105/tpc.109.071001 20511297 PMC2899873

[B9] Asano T. Yoshioka Y. Kurei S. Sakamoto W. Machida Y. (2004). A mutation of the CRUMPLED LEAF gene that encodes a protein localized in the outer envelope membrane of plastids affects the pattern of cell division, cell differentiation, and plastid division in Arabidopsis. Plant J. 38, 448–459. 10.1111/j.1365-313X.2004.02057.x 15086805

[B10] Awai K. Maréchal E. Block M. A. Brun D. Masuda T. Shimada H.. (2001). Two types of MGDG synthase genes, found widely in both 16: 3 and 18: 3 plants, differentially mediate galactolipid syntheses in photosynthetic and nonphotosynthetic tissues in *Arabidopsis thaliana*. Proc. Natl. Acad. Sci. U.S.A. 98, 10960–10965. 10.1073/pnas.181331498 11553816 PMC58581

[B11] Bahaji A. Li J. Ovecka M. Ezquer I. Muñoz F. J. Baroja-Fernández E.. (2011). *Arabidopsis thaliana* mutants lacking ADP-glucose pyrophosphorylase accumulate starch and wild-type ADP-glucose content: further evidence for the occurrence of important sources, other than ADP-glucose pyrophosphorylase, of ADP-glucose linked to leaf starch biosynthesis. Plant Cell Physiol. 52, 1162–1176. 10.1093/pcp/pcr067 21624897

[B12] Bauer J. Hiltbrunner A. Weibel P. Vidi P. A. Alvarez-Huerta M. Smith M. D.. (2002). Essential role of the G-domain in targeting of the protein import receptor atToc159 to the chloroplast outer membrane. J. Cell Biol. 159, 845–854. 10.1083/jcb.200208018 12460988 PMC2173394

[B13] Bauer M. Dietrich C. Nowak K. Sierralta W. D. Papenbrock J. (2004). Intracellular localization of Arabidopsis sulfurtransferases. Plant Physiol. 135, 916–926. 10.1104/pp.104.040121 15181206 PMC514126

[B14] Beardslee T. A. Roy-Chowdhury S. Jaiswal P. Buhot L. Lerbs-Mache S. Stern D. B.. (2002). A nucleus-encoded maize protein with sigma factor activity accumulates in mitochondria and chloroplasts. Plant J. 31, 199–209. 10.1046/j.1365-313X.2002.01344.x 12121449

[B15] Bédard J. Kubis S. Bimanadham S. Jarvis P. (2007). Functional similarity between the chloroplast translocon component, Tic40, and the human co-chaperone, Hsp70-interacting protein (Hip). J. Biol. Chem. 282, 21404–21414. 10.1074/jbc.M611545200 17535810

[B16] Belousov V. V. Fradkov A. F. Lukyanov K. A. Staroverov D. B. Shakhbazov K. S. Terskikh A. V.. (2006). Genetically encoded fluorescent indicator for intracellular hydrogen peroxide. Nat. Methods 3, 281–286. 10.1038/nmeth866 16554833

[B17] Block M. A. Jouhet J. (2015). Lipid trafficking at endoplasmic reticulum-chloroplast membrane contact sites. Curr. Opin. Cell Biol. 35, 21–29. 10.1016/j.ceb.2015.03.004 25868077

[B18] Bonzi L. M. Fabbri F. (1975). Chloroplast Protrusions in Arisarum Proboscideum (L.). Caryologica 28, 407–426. 10.1080/00087114.1975.10796628

[B19] Bourett T. M. Czymmek K. J. Howard R. J. (1999). Ultrastructure of chloroplast protuberances in rice leaves preserved by high-pressure freezing. Planta 208, 472–479. 10.1007/s004250050584

[B20] Breuers F. K. Bräutigam A. Geimer S. Welzel U. Y. Stefano G. Renna L.. (2012). Dynamic remodeling of the plastid envelope membranes - a tool for chloroplast envelope *in vivo* localizations. Front. Plant Sci. 3:7. 10.3389/fpls.2012.00007 22645566 PMC3355811

[B21] Breuers F. K. Bräutigam A. Weber A. P. (2011). The plastid outer envelope - a highly dynamic interface between Plastid and Cytoplasm. Front. Plant Sci. 2:97. 10.3389/fpls.2011.00097 22629266 PMC3355566

[B22] Briggs W. R. Beck C. F. Cashmore A. R. Christie J. M. Hughes J. Jarillo J. (2001). The phototropin family of photoreceptors. Plant Cell 13, 993–997. 10.1105/tpc.13.5.993 11424903 PMC1464709

[B23] Brunkard J. O. Runkel A. M. Zambryski P. C. (2015). Chloroplasts extend stromules independently and in response to internal redox signals. Proc. Natl. Acad. Sci. U.S.A. 112, 10044–10049. 10.1073/pnas.1511570112 26150490 PMC4538653

[B24] Buchanan B. B. Gruissem W. Jones R. L. (Eds.). (2015). Biochemistry and Molecular Biology of Plants, 2nd Edn. Toronto, ON: Wiley Blackwell.

[B25] Buchner O. Holzinger A. Lütz C. (2007a). Effects of temperature and light on the formation of chloroplast protrusions in leaf mesophyll cells of high alpine plants. Plant Cell Environ. 30, 1347–1356. 10.1111/j.1365-3040.2007.01707.x 17897406

[B26] Buchner O. Karadar M. Bauer I. Neuner G. (2013). A novel system for *in situ* determination of heat tolerance of plants: first results on alpine dwarf shrubs. Plant Methods 9:7. 10.1186/1746-4811-9-7 23497517 PMC3602033

[B27] Buchner O. Lütz C. Holzinger A. (2007b). Design and construction of a new temperature-controlled chamber for light and confocal microscopy under monitored conditions: biological application for plant samples. J. Microsc Oxford 225, 183–191. 10.1111/j.1365-2818.2007.01730.x 17359253

[B28] Buchner O. Stoll M. Karadar M. Kranner I. Neuner G. (2014). Application of heat stress *in situ* demonstrates a protective role of irradiation on photosynthetic performance in alpine plants. Plant Cell Environ. 38, 4. 10.1111/pce.12455 25256247 PMC4407927

[B29] Caplan J. L. Kumar A. S. Park E. Padmanabhan M. S. Hoban K. Modla S.. (2015). Chloroplast stromules function during innate immunity. Dev. Cell 34, 1–13. 10.1016/j.devcel.2015.05.011 26120031 PMC4596411

[B30] Caplan J. L. Mamillapalli P. Burch-Smith T. M. Czymmek K. Dinesh-Kumar S. P. (2008). Chloroplastic protein NRIP1 mediates innate immune receptor recognition of a viral effector. Cell 132, 449–462. 10.1016/j.cell.2007.12.031 18267075 PMC2267721

[B31] Chalfie M. Tu Y. Euskirchen G. Ward W. W. Prasher D. C. (1994). Green fluorescent protein as a marker for gene expression. Science 263, 802–805. 10.1126/science.8303295 8303295

[B32] Chew O. Whelan J. Millar A. H. (2003). Molecular definition of the ascorbate-glutathione cycle in *Arabidopsis* mitochondria reveals dual targeting of antioxidant defenses in plants. J. Biol. Chem. 278, 46869–46877. 10.1074/jbc.M307525200 12954611

[B33] Chi W. Feng P. Ma J. Zhang L. (2015). Metabolites and chloroplast retrograde signaling. Curr. Opin. Plant Biol. 25, 32–38. 10.1016/j.pbi.2015.04.006 25912815

[B34] Chiba A. Ishida H. Nishizawa N. K. Makino A. Mae T. (2003). Exclusion of ribulose-1, 5-bisphosphate carboxylase/oxygenase from chloroplasts by specific bodies in naturally senescing leaves of wheat. Plant Cell Physiol. 44, 914–921. 10.1093/pcp/pcg118 14519773

[B35] Chigri F. Sippel C. Kolb M. Vothknecht U. C. (2009). Arabidopsis OBG-like GTPase (AtOBGL) is localized in chloroplasts and has an essential function in embryo development. Mol. Plant 2, 1373–1383. 10.1093/mp/ssp073 19995735

[B36] Christiansen C. Hachem M. A. Glaring M. A. Viksø-Nielsen A. Sigurskjold B. W. Svensson B.. (2009). A CBM20 low-affinity starch-binding domain from glucan, water dikinase. FEBS Lett. 583, 1159–1163. 10.1016/j.febslet.2009.02.045 19275898

[B37] Comparot-Moss S. Kötting O. Stettler M. Edner C. Graf A. Weise S. E.. (2010). A putative phosphatase, LSF1, is required for normal starch turnover in Arabidopsis leaves. Plant Physiol. 152, 685–697. 10.1104/pp.109.148981 20018601 PMC2815883

[B38] Costa A. Drago I. Behera S. Zottini M. Pizzo P. Schroeder J. I. (2010). H2O2 in plant peroxisomes: an *in vivo* analysis uncovers a Ca2+-dependent scavenging system. Plant J. 62, 760–772. 10.1111/j.1365-313X.2010.04190.x 20230493 PMC2884081

[B39] Delatte T. Umhang M. Trevisan M. Eicke S. Thorneycroft D. Smith S. M.. (2006). Evidence for distinct mechanisms of starch granule breakdown in plants. J. Biol. Chem. 281, 12050–12059. 10.1074/jbc.M513661200 16495218

[B40] Dinkins R. D. Conn H. M. Dirk L. M. A. Williams M. A. Houtz R. L. (2003). The *Arabidopsis thaliana* peptide deformylase 1 protein is localized to both mitochondria and chloroplasts. Plant Sci. 165, 751–758. 10.1016/S0168-9452(03)00236-X

[B41] Dupree P. Pwee K. H. Gray J. C. (1991). Expression of photosynthesis gene-promoter fusions in leaf epidermal cells of transgenic tobacco plants. Plant J. 1, 115–120. 10.1111/j.1365-313X.1991.00115.x

[B42] Erickson J. L. Ziegler J. Guevara D. Abel S. Klösgen R. B. Mathur J.. (2014). Agrobacterium-derived cytokinin influences plastid morphology and starch accumulation in *Nicotiana benthamiana* during transient assays. BMC Plant Biol. 14:127. 10.1186/1471-2229-14-127 24886417 PMC4062310

[B43] Farmaki T. Sanmartín M. Jiménez P. Paneque M. Sanz C. Vancanneyt G.. (2007). Differential distribution of the lipoxygenase pathway enzymes within potato chloroplasts. J. Exp. Bot. 58, 555–568. 10.1093/jxb/erl230 17210991

[B44] Fernández A. P. Strand Å. (2008). Retrograde signaling and plant stress: plastid signals initiate cellular stress responses. Curr. Opin. Plant Biol. 11, 509–513. 10.1016/j.pbi.2008.06.002 18639482

[B45] Ferro M. Brugière S. Salvi D. Seigneurin-Berny D. Moyet L. Ramus C.. (2010). AT_CHLORO, a comprehensive chloroplast proteome database with subplastidial localization and curated information on envelope proteins. Mol. Cell. Proteomics 9, 1063–1084. 10.1074/mcp.M900325-MCP200 20061580 PMC2877971

[B46] Ferro M. Salvi D. Rivière-Rolland H. Vermat T. Seigneurin-Berny D. Grunwald D.. (2002). Integral membrane proteins of the chloroplast envelope: identification and subcellular localization of new transporters. Proc. Natl. Acad. Sci. U.S.A. 99, 11487–11492. 10.1073/pnas.172390399 12177442 PMC123283

[B47] Fester T. Strack D. Hause B. (2001). Reorganization of tobacco root plastids during arbuscule development. Planta 213, 864–868. 10.1007/s004250100561 11722122

[B48] Foyer C. H. Noctor G. (2009). Redox regulation in photosynthetic organisms: signaling, acclimation, and practical implications. Antioxid. Redox Sign. 11, 861–905. 10.1089/ars.2008.2177 19239350

[B49] Fujiwara M. T. Hashimoto H. Kazama Y. Abe T. Yoshida S. (2008). The assembly of the FtsZ ring at the mid-chloroplast division site depends on a balance between the activities of AtMinE1 and ARC11/AtMinD1. Plant Cell Physiol. 49, 345–361. 10.1093/pcp/pcn012 18204083

[B50] Gámez-Arjona F. M. de la Concepción J. C. Raynaud S. Mérida Á. (2014b). *Arabidopsis thaliana* plastoglobule-associated fibrillin 1a interacts with fibrillin 1b *in vivo*. FEBS Lett. 588, 2800–2804. 10.1016/j.febslet.2014.06.024 24937144

[B51] Gámez-Arjona F. M. Raynaud S. Ragel P. Mérida Á. (2014a). Starch synthase 4 is located in the thylakoid membrane and interacts with plastoglobule−associated proteins in Arabidopsis. Plant J. 80, 305–316. 10.1111/tpj.12633 25088399

[B52] Gao H. Kadirjan-Kalbach D. Froehlich J. E. Osteryoung K. W. (2003). ARC5, a cytosolic dynamin-like protein from plants, is part of the chloroplast division machinery. Proc. Natl. Acad. Sci. U.S.A. 100, 4328–4333. 10.1073/pnas.0530206100 12642673 PMC153092

[B53] Glińska S. Gapińska M. Michlewska S. Skiba E. Kubicki J. (2015). Analysis of Triticum aestivum seedling response to the excess of zinc. Protoplasma. 10.1007/s00709-015-0816-3. [Epub ahead of print]. 25902894 PMC4783454

[B54] Glynn J. M. Froehlich J. E. Osteryoung K. W. (2008). Arabidopsis ARC6 coordinates the division machineries of the inner and outer chloroplast membranes through interaction with PDV2 in the intermembrane space. Plant Cell 20, 2460–2470. 10.1105/tpc.108.061440 18812496 PMC2570736

[B55] Glynn J. M. Yang Y. Vitha S. Schmitz A. J. Hemmes M. (2009). PARC6, a novel chloroplast division factor, influences FtsZ assembly and is required for recruitment of PDV1 during chloroplast division in Arabidopsis. Plant J. 59, 700–711. 10.1111/j.1365-313X.2009.03905.x 19453460

[B56] Gray J. C. Hansen M. R. Shaw D. J. Graham K. Dale R. Smallman P. (2012). Plastid stromules are induced by stress treatments acting through abscisic acid. Plant J. 69, 387–398. 10.1111/j.1365-313X.2011.04800.x 21951173

[B57] Gray J. C. Sullivan J. A. Hibberd J. M. Hanson M. R. (2001). Stromules: mobile protrusions and interconnections between plastids. Plant Biol. 3, 223–233. 10.1055/s-2001-15204

[B58] Green P. B. (1964). Cinematic observations on the growth and division of chloroplasts in Nitella. Am. J. Bot. 51, 334–342. 10.2307/2440306

[B59] Gunning B. E. S. (1965). The greening process in plastids. Protoplasma 60, 111–130. 10.1007/BF01248133

[B60] Gunning B. E. S. (2001). Membrane geometry of “open” prolamellar bodies. Protoplasma 215, 4–15. 10.1007/BF01280299 11732064

[B61] Gunning B. E. S. (2005). Plastid stromules: video microscopy of their outgrowth, retraction, tensioning, anchoring, branching, bridging, and tip-shedding. Protoplasma 225, 33–42. 10.1007/s00709-004-0073-3 15868211

[B62] Gunning B. E. S. Koenig F. Govindjee G. (2007). A dedication to pioneers of research on chloroplast structure. XXIII, in The Structure and Function of Plastids, eds Wise R. R. Hoober J. K. (Dordrecht: Springer), xxiii–xxxi.

[B63] Gutman B. L. Niyogi K. K. (2009). Evidence for base excision repair of oxidative DNA damage in chloroplasts of *Arabidopsis thaliana*. J. Biol. Chem. 284, 17006–17012. 10.1074/jbc.M109.008342 19372224 PMC2719338

[B64] Hans J. Hause B. Strack D. Walter M. H. (2004). Cloning, characterization, and immunolocalization of a mycorrhiza-inducible 1-deoxy-d-xylulose 5-phosphate reductoisomerase in arbuscule-containing cells of maize. Plant Physiol. 13, 614–624. 10.1104/pp.103.032342 14764905 PMC344538

[B65] Hanson G. T. Aggeler R. Oglesbee D. Cannon M. Capaldi R. A. Tsien R. Y.. (2004). Investigating mitochondrial redox potential with redox-sensitive green fluorescent protein indicators. J. Biol. Chem. 279, 13044–13053. 10.1074/jbc.M312846200 14722062

[B66] Hanson M. R. Köhler R. H. (2006). A novel view of chloroplast structure. Web Essay: 7.1, in Plant Physiology, eds Taiz V. L. Zeiger E. (Sunderland: Sinauer Associates).

[B67] Hanson M. R. Sattarzadeh A. (2008). Dynamic morphology of plastids and stromules in angiosperm plants. Plant Cell Environ. 31, 646–657. 10.1111/j.1365-3040.2007.01768.x 18088332

[B68] Hanson M. R. Sattarzadeh A. (2011). Stromules: recent insights into a long neglected feature of plastid morphology and function. Plant Physiol. 155, 1486–1492. 10.1104/pp.110.170852 21330493 PMC3091082

[B69] Hanson M. R. Sattarzadeh A. (2013). Trafficking of proteins through plastid stromules. Plant Cell 25, 2774–2782. 10.1105/tpc.113.112870 23983219 PMC3784579

[B70] Hara T. Katoh H. Ogawa D. Kagaya Y. Sato Y. Kitano H.. (2015). Rice SNF2 family helicase ENL1 is essential for syncytial endosperm development. Plant J. 81, 1–12. 10.1111/tpj.12705 25327517

[B71] Harris J. B. Arnott H. J. (1973). Effects of senescence on chloroplasts of the tobacco leaf. Tissue Cell 5, 527–544. 10.1016/S0040-8166(73)80043-6 4768400

[B72] Haseloff J. Siemering K. R. Prasher D. C. Hodge S. (1997). Removal of a crypic intron and subcellular localization of green fluorescent protein are required to mark transgenic Arabidopsis plants brightly. Proc. Natl. Acad. Sci. U.S.A. 94, 2122–2127. 10.1073/pnas.94.6.2122 9122158 PMC20051

[B73] Helliwell C. A. Sullivan J. A. Mould R. M. Gray J. C. Peacock W. J. Dennis E. S. (2001). A plastid envelope location of Arabidopsis ent-kaurene oxidase links the plastid and endoplasmic reticulum steps of the gibberellin biosynthesis pathway. Plant J. 28, 201–208. 10.1046/j.1365-313X.2001.01150.x 11722763

[B74] Holzinger A. Buchner O. Lütz C. and Hanson, M. R. (2007b). Temperature-sensitive formation of chloroplast protrusions and stromules in mesophyll cells of *Arabidopsis thaliana*. Protoplasma 230, 23–30. 10.1007/s00709-006-0222-y 17351732

[B75] Holzinger A. Kwok E. Y. Hanson M. R. (2008). Effects of arc3, arc5 and arc6 mutations on plastid morphology and stromule formation in green and nongreen tissues of *Arabidopsis thaliana*. Photochem. Photobiol. 84, 1324–1335. 10.1111/j.1751-1097.2008.00437.x 18764889

[B76] Holzinger A. Wasteneys G. O. Lütz C. (2007a). Investigating cytoskeletal function in chloroplast protrusion formation in the arctic-alpine plant Oxyria digyna. Plant Biol. 9, 400–410. 10.1055/s-2006-924727 17236103

[B77] Hoober J. K. (2007). Chloroplast development: whence and whither. Chapter 2, in The Structure and Function of Plastids, eds Wise R. R. Hoober J. K. (Dordrecht: Springer), 27–51.

[B78] Ishida H. Izumi M. Wada S. Makino A. (2014). Roles of autophagy in chloroplast recycling. Biochim. Biophys. Acta. 1837, 512–521. 10.1016/j.bbabio.2013.11.009 24269172

[B79] Ishida H. Yoshimoto K. Izumi M. Reisen D. Yano Y. Makino A.. (2008). Mobilization of rubisco and stroma-localized fluorescent proteins of chloroplasts to the vacuole by an ATG gene-dependent autophagic process. Plant Physiol. 148, 142–155. 10.1104/pp.108.122770 18614709 PMC2528122

[B80] Itoh R. D. Yamasaki H. Septiana A. Yoshida S. Fujiwara M. T. (2010). Chemical induction of rapid and reversible plastid filamentation in *Arabidopsis thaliana* roots. Physiol. Plant. 139, 144–158. 10.1111/j.1399-3054.2010.01352.x 20088905

[B81] Izumi M. Hidema J. Ishida H. (2015). From *Arabidopsis* to cereal crops: Conservation of chloroplast protein degradation by autophagy indicates its fundamental role in plant productivity. Plant Signal. Behav. 10:e1101199. 10.1080/15592324.2015.1101199 26440746 PMC4883919

[B82] Jaipargas E. A. (2015). Investigations on Mitochondrial Pleomorphy and Interactions with the Endoplasmic Reticulum and Peroxisomes. Diss. University of Guelph, Guelph.

[B83] Jeske H. Werz G. (1978). The influence of light intensity on pigment composition and ultrastructure of plastids in leaves of diseased *Abutilon sellowianum* Reg. (Malvaceae). J. Phytopathol. 91, 1–13. 10.1111/j.1439-0434.1978.tb04189.x

[B84] Jiang K. Schwarzer C. Lally E. Zhang S. Ruzin S. Machen T.. (2006). Expression and characterization of a redox-sensing green fluorescent protein (reduction-oxidation-sensitive green fluorescent protein) in Arabidopsis. Plant Physiol. 141, 397–403. 10.1104/pp.106.078246 16760494 PMC1475439

[B85] Joo J. H. Wang S. Chen J. G. Jones A. M. Fedoroff N. V. (2005). Different signaling and cell death roles of heterotrimeric G protein alpha and beta subunits in the Arabidopsis oxidative stress response to ozone. Plant Cell 17, 957–970. 10.1105/tpc.104.029603 15705948 PMC1069711

[B86] Jouhet J. Maréchal E. Baldan B. Bligny R. Joyard J. Block M. A. (2004). Phosphate deprivation induces transfer of DGDG galactolipid from chloroplast to mitochondria. J. Cell Biol. 167, 863–874. 10.1083/jcb.200407022 15569715 PMC2172463

[B87] Kadota A. Yamada N. Suetsugu N. Hirose M. Saito C. Shoda K.. (2009). Short actin-based mechanism for light-directed chloroplast movement in Arabidopsis. Proc. Natl. Acad. Sci. U.S.A. 106, 13106–13111. 10.1073/pnas.0906250106 19620714 PMC2722281

[B88] Kagawa T. Kasahara M. Abe T. Yoshida S. Wada M. (2004). Function analysis of phototropin2 using fern mutants deficient in blue light-induced chloroplast avoidance movement. Plant Cell Physiol. 45, 416–426. 10.1093/pcp/pch045 15111716

[B89] Kandasamy M. K. Meagher R. B. (1999). Actin-organelle interaction: association with chloroplast in Arabidopsis leaf mesophyll cells. Cell Motil. Cytoskeleton 44, 110–118. 10506746 10.1002/(SICI)1097-0169(199910)44:2<110::AID-CM3>3.0.CO;2-O

[B90] Kasahara M. Kagawa T. Oikawa K. Suetsugu N. Miyao M. Wada M. (2002). Chloroplast avoidance movement reduces photodamage in plants. Nature 420, 829–832. 10.1038/nature01213 12490952

[B91] Kasahara M. Kagawa T. Sato Y. Kiyosue T. Wada M. (2004). Phototropins mediate blue and red light-induced chloroplast movements in Physcomitrella patens. Plant Physiol. 135, 1388–1397. 10.1104/pp.104.042705 15247376 PMC519056

[B92] Kasmati A. R. Töpel M. Patel R. Murtaza G. Jarvis P. (2011). Molecular and genetic analyses of Tic20 homologues in *Arabidopsis thaliana* chloroplasts. Plant J. 66, 877–889. 10.1111/j.1365-313X.2011.04551.x 21395885

[B93] Kiessling J. Martin A. Gremillon L. Rensing S. A. Nick P. Sarnighausen E.. (2004). Dual targeting of plastid division protein FtsZ to chloroplasts and the cytoplasm. EMBO Reports 5, 889–894. 10.1038/sj.embor.7400238 15319781 PMC1299139

[B94] Kim C. Apel K. (2004). Substrate-dependent and organ-specific chloroplast protein import in planta. Plant Cell 16, 88–98. 10.1105/tpc.015008 14688290 PMC301397

[B95] Kim C. Meskauskiene R. Zhang S. Lee K. P. Ashok M. L. Blajecka K.. (2012). Chloroplasts of Arabidopsis are the source and a primary target of a plant-specific programmed cell death signaling pathway. Plant Cell 24, 3026–3039. 10.1105/tpc.112.100479 22797473 PMC3426130

[B96] Kitajima A. Asatsuma S. Okada H. Hamada Y. Kaneko K. Nanjo Y.. (2009). The rice α-amylase glycoprotein is targeted from the golgi apparatus through the secretory pathway to the plastids. Amer. Soc. Plant Biol. 21, 2844–2858. 10.1105/tpc.109.068288 19767453 PMC2768910

[B97] Köhler R. H. Cao J. Zipfel W. R. Webb W. W. Hanson M. R. (1997). Exchange of protein molecules through connections between higher plant plastids. Science 276, 2039–2042. 10.1126/science.276.5321.2039 9197266

[B98] Köhler R. H. Hanson M. R. (2000). Plastid tubules of higher plants are tissue-specific and developmentally regulated. J. Cell Sci. 113, 81–89. 10591627 10.1242/jcs.113.1.81

[B99] Kölling K. Thalmann M. Müller A. Jenny C. Zeeman S. C. (2015). Carbon partitioning in *Arabidopsis thaliana* is a dynamic process controlled by the plants metabolic status and its circadian clock. Plant Cell Environ. 38, 1965–1979. 10.1111/pce.12512 25651812 PMC4671261

[B100] Kong S.-G. Wada M. (2011). New insights into dynamic actin-based chloroplast photorelocation movement. Mol. Plant 4, 771–781. 10.1093/mp/ssr061 21772030

[B101] Kost B. Spielhofer P. Chua N.-H. (1998). A GFP-mouse talin fusion protein labels plant actin filaments *in vivo* and visualizes the actin cytoskeleton in growing pollen tubes. Plant J. 16, 393–401. 10.1046/j.1365-313x.1998.00304.x 9881160

[B102] Krenz B. Guo T. W. Kleinow T. (2014). Stromuling when stressed! Acta. Soc. Bot. Pol. 83, 325–329. 10.5586/asbp.2014.05025805692

[B103] Krenz B. Jeske H. Kleinow T. (2012). The induction of stromule formation by a plant DNA-virus in epidermal leaf tissues suggests a novel intra- and intercellular macromolecular trafficking route. Front. Plant Sci. 3:291. 10.3389/fpls.2012.00291 23293643 PMC3530832

[B104] Krenz B. Windeisen V. Wege C. Jeske H. Kleinow T. (2010). A plastid-targeted heat shock cognate 70 kDa protein interacts with the Abutilon mosaic virus movement protein. Virology 401, 6–17. 10.1016/j.virol.2010.02.011 20193958

[B105] Krishnakumar V. Choi Y. Beck E. Wu Q. Luo A. Sylvester A.. (2015). A maize database resource that captures tissue-specific and subcellular-localized gene expression, via fluorescent tags and confocal imaging (Maize Cell Genomics Database). Plant Cell Physiol. 56, e12. 10.1093/pcp/pcu178 25432973

[B106] Krupinska K. (2007). Fate and activities of plastids during leaf senescence, in The Structure and Function of Plastids, eds Wise R. R. Hoober J. K. Govindjeee G. (Dordrecht: Springer), 433–449.

[B107] Kwok E. Y. Hanson M. R. (2004a). GFP-labelled Rubisco and aspartate aminotransferase are present in plastid stromules and traffic between plastids. J. Exp. Bot. 55, 595–604. 10.1093/jxb/erh062 14754918

[B108] Kwok E. Y. Hanson M. R. (2004b). Plastids and stromules interact with the nucleus and cell membrane in vascular plants. Plant Cell Rep. 23, 188–195. 10.1007/s00299-004-0824-9 15252692

[B109] Kwok E. Y. Hanson M. R. (2004c). *In vivo* analysis of interactions between GFP-labeled microfilaments and plastid stromules. BMC Plant Biol. 4:2. 10.1186/1471-2229-4-2 15018639 PMC356911

[B110] Kwok E. Y. Hanson M. R. (2004d). Stromules and the dynamic nature of plastid morphology. J. Microsc. 214, 124–137. 10.1111/j.0022-2720.2004.01317.x 15102061

[B111] Kwok R. H. Hanson M. R. (2003). Microtubules and microfilaments control the morphology and movement of non-green plastids and stromules in *Nicotiana tabacum*. Plant J. 35, 16–26. 10.1046/j.1365-313X.2003.01777.x 12834398

[B112] Larcher W. Wagner J. Lütz C. (1997). The effect of heat on photosynthesis, dark respiration and cellular ultrastructure of the arctic-alpine psychrophyte *Ranunculus glacialis*. Photosynthetica 34, 219–232. 10.1023/A:1006840623763

[B113] Lee Y. J. Kim D. H. Kim Y. W. Hwang I. (2001). Identification of a signal that distinguishes between the chloroplast outer envelope membrane and the endomembrane system *in vivo*. Plant Cell 13, 2175–2190. 10.1105/tpc.13.10.2175 11595795 PMC139152

[B114] Leeuwenhoek A. V (1674). Letter 11/6 in Phil Trans Vol IX-108. London. Available online at: http://www.dbnl.org/tekst/leeu027alle01_01/leeu027alle01_01_0013.php#b0011

[B115] Lehmann P. Bohnsack M. T. Schleiff E. (2011). The functional domains of the chloroplast unusual positioning protein 1. Plant Sci. 180, 650–654. 10.1016/j.plantsci.2011.01.006 21421414

[B116] Levitan A. Trebitsh T. Kiss V. Pereg Y. Dangoor I. Danon A. (2005). Dual targeting of the protein disulfide isomerase RB60 to chloroplast and the endoplasmic reticulum. Proc. Natl. Acad. Sci. U.S.A. 102, 6225–6230. 10.1073/pnas.0500676102 15837918 PMC1087927

[B117] Lichtenthaler H. K. (2013). Plastoglobuli, thylakoids, chloroplast structure and development of plastids, in Plastid Development in Leaves During Growth and Senescence, Advances in Photosynthesis and Respiration, eds Biswal B. Krupinska K. Biswal U. C. (Dordrecht: Springer), 337–361. 10.1007/978-94-007-5724-0_15

[B118] Logan D. C. Leaver C. J. (2000). Mitochondria-targeted GFP highlights the heterogeneity of mitochondrial shape, size and movement within living plant cells. J. Exp. Bot. 51, 865–871. 10.1093/jexbot/51.346.865 10948212

[B119] Lohse S. Schliemann W. Ammer C. Kopka J. Strack D. Fester T. (2005). Organization and metabolism of plastids and mitochondria in arbuscular mycorrhizal roots of Medicago truncatula. Plant Physiol. 139, 329–340. 10.1104/pp.105.061457 16126866 PMC1203382

[B120] Lütz C. (1987). Cytology of high alpine plants II. Microbody activity in leaves of Ranunculus glacialis L. Cytologia 52, 679–686. 10.1508/cytologia.52.679

[B121] Lütz C. (2010). Cell physiology of plants growing in cold environments. Protoplasma 244, 53–73. 10.1007/s00709-010-0161-5 20521070

[B122] Lütz C. Bergweiler P. Di Piazza L. Holzinger A. (2012). Cell organelle structure and function in Alpine and Polar plants are influenced by growth conditions and climate, in Plants in Alpine Regions. Cell Physiology of Adaption and Survival Strategies, ed Lütz C. (Vienna: Springer), 43–60. 10.1007/978-3-7091-0136-0_5

[B123] Lütz C. Engel L. (2007). Changes in chloroplast ultrastructure in some high-alpine plants: adaptation to metabolic demands and climate? Protoplasma 231, 183–192. 10.1007/s00709-007-0249-8 17603748

[B124] Lütz C. Moser W. (1977). Beiträge zur cytologie hochalpiner Pflanzen. I. Untersuchungen zur Ultrastruktur von Ranunculus glacialis L. Flora 166, 21–34.

[B125] Mano S. Miwa T. Nishikawa S. Mimura T. Nishimura M. (2011). The Plant Organelles Database 2 (PODB2): an updated resource containing movie data of plant organelle dynamics. Plant Cell Physiol. 52, 244–253. 10.1093/pcp/pcq184 21115470 PMC3037075

[B126] Maple J. Fujiwara M. T. Kitahata N. Lawson T. Baker N. R. Yoshida S.. (2004). GIANT CHLOROPLAST 1 is essential for correct plastid division in Arabidopsis. Curr. Biol. 14, 776–781. 10.1016/j.cub.2004.04.031 15120068

[B127] Maple J. Vojta L. Soll J. Møller S. G. (2007). ARC3 is a stromal Z−ring accessory protein essential for plastid division. EMBO Rep. 8, 293–299. 10.1038/sj.embor.7400902 17304239 PMC1808034

[B128] Margulis L. (1970). Origin of the Eukaryotic Cells. New Haven, CT: Yale University Press.

[B129] Marques J. P. Schattat M. H. Hause G. Dudeck I. Klösgen R. B. (2004). *In vivo* transport of folded EGFP by the ΔpH/TAT-dependent pathway in chloroplasts of *Arabidopsis thaliana*. J. Exp. Bot. 55, 1697–1706. 10.1093/jxb/erh191 15208333

[B130] Mathur J. (2007). The illuminated plant cell. Trends Plant Sci. 12, 506–513. 10.1016/j.tplants.2007.08.017 17933577

[B131] Mathur J. Barton K. A. Schattat M. H. (2013). Fluorescent protein flow within stromules. Plant Cell 25, 2771–2772. 10.1105/tpc.113.117416 23983222 PMC3784578

[B132] Mathur J. Mammone A. Barton K. A. (2012). Organelle extensions in plant cells. J. Integr. Plant Biol. 54, 851–867. 10.1111/j.1744-7909.2012.01175.x 23046073

[B133] Mathur J. Radhamony R. Sinclair A. M. Donoso A. Dunn N. Roach E. Mathur, N.. (2010). mEosFP-based green-to-red photoconvertible subcellular probes for plants. Plant Physiol. 154, 1573–1587. 10.1104/pp.110.165431 20940350 PMC2996014

[B134] Matsushima R. Maekawa M. Kusano M. Kondo H. Fujita N. Kawagoe Y.. (2014). Amyloplast-localized SUBSTANDARD STARCH GRAIN4 protein influences the size of starch grains in rice endosperm. Plant Physiol. 164, 623–636. 10.1104/pp.113.229591 24335509 PMC3912094

[B135] McLean B. Whatley J. M. Juniper B. E. (1988). Continuity of chloroplast and endoplasmic-reticulum mem- branes in chara and equisetum. New Phytol. 109, 59–65. 10.1111/j.1469-8137.1988.tb00219.x

[B136] Melonek J. Matros A. Trösch M. Mock H. P. Krupinska K. (2012). The core of chloroplast nucleoids contains architectural SWIB domain proteins. Plant Cell 24, 3060–3073. 10.1105/tpc.112.099721 22797472 PMC3426132

[B137] Menke W. (1962). Structure and chemistry of plastids. Annu. Rev. Plant Physiol. 13, 27–44. 10.1146/annurev.pp.13.060162.000331

[B138] Menzel D. (1994). An interconnected plastidom in Acetabularia: implica- tions for the mechanism of chloroplast motility. Protoplasma 179, 166–171. 10.1007/BF01403955

[B139] Meyer A. J. Brach T. Marty L. Kreye S. Rouhier N. Jacquot J. P.. (2007). Redox-sensitive GFP in *Arabidopsis thaliana* is a quantitative biosensor for the redox potential of the cellular glutathione redox buffer. Plant J. 52, 973–986. 10.1111/j.1365-313X.2007.03280.x 17892447

[B140] Miras S. Salvi D. Ferro M. Grunwald D. Garin J. Joyard J.. (2002). Non-canonical transit peptide for import into the chloroplast. J. Biol. Chem. 277, 47770–47778. 10.1074/jbc.M207477200 12368288

[B141] Miyagishima S. Y. Froehlich J. E. Osteryoung K. W. (2006). PDV1 and PDV2 mediate recruitment of the dynamin-related protein ARC5 to the plastid division site. Plant Cell 18, 2517–2530. 10.1105/tpc.106.045484 16998069 PMC1626610

[B142] Moser T. Holzinger A. Buchner O. (2015). Chloroplast protrusions in leaves of Ranunculus glacialis L. respond significantly to different ambient conditions but are not related to temperature stress. Plant Cell Environ. 38, 7 10.1111/pce.12483 25393014 PMC5098225

[B143] Mueller S. J. Lang D. Hoernstein S. N. Lang E. G. Schuessele C. Schmidt A.. (2014). Quantitative analysis of the mitochondrial and plastid proteomes of the moss Physcomitrella patens reveals protein macrocompartmentation and microcompartmentation. Plant Physiol. 164, 2081–2095. 10.1104/pp.114.235754 24515833 PMC3982764

[B144] Muñoz F. J. Baroja-Fernández E. Ovecka M. Li J. Mitsui T. Sesma M. T.. (2008). Plastidial localization of a potato ‘Nudix’ hydrolase of ADP-glucose linked to starch biosynthesis. Plant Cell Physiol. 49, 1734–1746. 10.1093/pcp/pcn145 18801762

[B145] Murayama S. Handa H. (2007). Genes for alkaline/neutral invertase in rice: alkaline/neutral invertases are located in plant mitochondria and also in plastids. Planta 225, 1193–1203. 10.1007/s00425-006-0430-x 17086397

[B146] Myouga F. Hosoda C. Umezawa T. Izumi H. Kuromori T. Motohashi R.. (2008). A heterocomplex of iron superoxide dismutases defends chloroplast nucleoids against oxidative stress and is essential for chloroplast development in Arabidopsis. Plant Cell 20, 3148–3162. 10.1105/tpc.108.061341 18996978 PMC2613658

[B147] Nacir H. Bréhélin C. (2013). When proteomics reveals unsuspected roles: the plastoglobule example. Front. Plant Sci. 4:114. 10.3389/fpls.2013.00114 23630540 PMC3635846

[B148] Nakamura T. Yamaguchi Y. Sano H. (2000). Plant mercaptopyruvate sulfurtransferases. Eur. J. Biochem. 267, 5621–5630. 10.1046/j.1432-1327.2000.01633.x 10951223

[B149] Nakanishi H. Suzuki K. Kabeya Y. Miyagishima S. (2009). Plant-specific protein MCD1 determines the site of chloroplast division in concert with bacterial derived MinD. Curr. Biol. 27, 151–156. 10.1016/j.cub.2008.12.018 19135368

[B150] Natesan S. K. Sullivan J. A. Gray J. (2005). Stromules: a characteristic cell- specific feature of plastid morphology. J. Exp. Bot. 56, 787–797. 10.1093/jxb/eri088 15699062

[B151] Natesan S. K. Sullivan J. A. Gray J. C. (2009). Myosin XI is required for actin-associated movement of plastid stromules. Mol. Plant. 2, 1262–1272. 10.1093/mp/ssp078 19995729

[B152] Newell C. A. Natesan S. K. Sullivan J. A. Jouhet J. Kavanagh T. A. Gray J. C. (2012). Exclusion of plastid nucleoids and ribosomes from stromules in tobacco and Arabidopsis. Plant J. 69, 399–410. 10.1111/j.1365-313X.2011.04798.x 21951134

[B153] Nobusawa T. Umeda M. (2012). Very−long−chain fatty acids have an essential role in plastid division by controlling Z−ring formation in *Arabidopsis thaliana*. Genes Cells 17, 709–719. 10.1111/j.1365-2443.2012.01619.x 22734690

[B154] Oikawa K. Kasahara M. Kiyosue T. Kagawa T. Suetsugu N. Takahashi F.. (2003). CHLOROPLAST UNUSUAL POSITIONING1 is essential for proper chloroplast positioning. Plant Cell 15, 2805–2815. 10.1105/tpc.016428 14615600 PMC282804

[B155] Oikawa K. Yamasato A. Kong S.-G. Kasahara M. Nakai M. Takahashi F.. (2008). Chloroplast outer envelope protein CHUP1 is essential for chloroplast anchorage to the plasma membrane and chloroplast movement. Plant Physiol. 148, 829–842. 10.1104/pp.108.123075 18715957 PMC2556824

[B156] Osteryoung K. W. Pyke K. A. (2014). Division and dynamic morphology of plastids. Annu. Rev. Plant. Biol. 65, 443–472. 10.1146/annurev-arplant-050213-035748 24471836

[B157] Paves H. Truve E. (2007). Myosin inhibitors block accumulation movement of chloroplasts in *Arabidopsis thaliana* leaf cells. Protoplasma 230, 165–169. 10.1007/s00709-006-0230-y 17458631

[B158] Piller L. E. Besagni C. Ksas B. Rumeau D. Bréhélin C. Glauser G.. (2011). Chloroplast lipid droplet type II NAD (P) H quinone oxidoreductase is essential for prenylquinone metabolism and vitamin K1 accumulation. Proc. Natl. Acad. Sci. U.S.A. 108, 14354–14359. 10.1073/pnas.1104790108 21844348 PMC3161580

[B159] Primavesi L. F. Wu H. Mudd E. A. Day A. Jones H. D. (2008). Visualisation of plastids in endosperm, pollen and roots of transgenic wheat expressing modified GFP fused to transit peptides from wheat SSU RubisCO, rice FtsZ and maize ferredoxin III proteins. Transgen. Res. 17, 529–543. 10.1007/s11248-007-9126-7 17710559

[B160] Puthiyaveetil S. Kavanagh T. A. Cain P. Sullivan J. A. Newell C. A. Gray J. C.. (2008). The ancestral symbiont sensor kinase CSK links photosynthesis with gene expression in chloroplasts. Proc. Natl. Acad. Sci. U.S.A. 105, 10061–10066. 10.1073/pnas.0803928105 18632566 PMC2474565

[B161] Pyke K. A. (2009). Plastid Biology. Cambridge: Cambridge University Press.

[B162] Pyke K. A. Howells C. A. (2002). Plastid and stromule morphogenesis in tomato. Ann. Bot. 90, 559–566. 10.1093/aob/mcf235 12466096 PMC4240451

[B163] Qiao J. Ma C. Wimmelbacher M. Börnke F. Luo M. (2011). Two novel proteins, MRL7 and its paralog MRL7-L, have essential but functionally distinct roles in chloroplast development and are involved in plastid gene expression regulation in Arabidopsis. Plant Cell Physiol. 52, 1017–1030. 10.1093/pcp/pcr054 21515910

[B164] Robertson E. J. Rutherford S. M. Leech R. M. (1996). Characterization of chloroplast division using the Arabidopsis mutant arc5. Plant Physiol. 112, 149–159. 10.1104/pp.112.1.149 8819323 PMC157934

[B165] Ruberti C. Costa A. Pedrazzini E. Schiavo F. L. Zottini M. (2014). FISSION1A, an *Arabidopsis* tail-anchored protein, is localized to three subcellular compartments. Mol. Plant 7, 1393–1396. 10.1093/mp/ssu027 24658461

[B166] Sage T. L. Sage R. F. (2009). The functional anatomy of rice leaves: implications for refixation of photorespiratory CO2 and efforts to engineer C4 Photosynthesis into rice. Plant Cell Physiol. 50, 756–772. 10.1093/pcp/pcp033 19246459

[B167] Sakai T. Kagawa T. Kasahara M. Swartz T. E. Christie J. M. Briggs W. R. (2001). Arabidopsis nph1 and npl1: blue light receptors that mediate both phototropism and chloroplast relocation. Proc. Natl. Acad. Sci. U.S.A. 98, 6969–6974. 10.1073/pnas.101137598 11371609 PMC34462

[B168] Sandalio L. M. Foyer C. H. (2015). Unravelling the reactive oxygen and reactive nitrogen signalling networks in plants. J. Expt. Bot. 66, 2825–2826. 10.1093/jxb/erv124 26151076 PMC4423514

[B169] Sato N. (2007). Origin and evolution of plastids: genomic view on the unification and diversity of plastids chapter 4, in The Structure and Function of Plastids, eds Wise R. R. Hoober J. K. (Dordrecht: Springer), 75–102.

[B170] Sattarzadeh A. Krahmer J. Germain A. D. Hanson M. R. (2009). A myosin XI tail domain homologous to the yeast myosin vacuole-binding domain interacts with plastids and stromules in *Nicotiana benthamiana*. Mol. Plant. 2, 1351–1358. 10.1093/mp/ssp094 19995734

[B171] Schattat M. H. Barton K. A. Mathur J. (2015). The myth of interconnected plastids and related phenomena. Protoplasma 252, 359–371. 10.1007/s00709-014-0666-4 24965372

[B172] Schattat M. H. Barton K. Baudisch B. Klösgen R. B. Mathur J. (2011a). Plastid stromule branching coincides with contiguous endoplasmic reticulum dynamics. Plant Physiol. 155, 1667–1677. 10.1104/pp.110.170480 21273446 PMC3091094

[B173] Schattat M. H. Barton K. Mathur J. (2011b). Correlated behavior implicates stromules in increasing the interactive surface between plastids and ER tubules. Plant Signal. Behav. 6, 715–718. 10.4161/psb.6.5.15085 21448009 PMC3172846

[B174] Schattat M. H. Griffiths S. Mathur N. Barton K. Wozny M. R. Dunn N. (2012a). Differential coloring reveals that plastids do not form networks for exchanging macromolecules. Plant Cell 24, 1465–1477. 10.1105/tpc.111.095398 22474180 PMC3398557

[B175] Schattat M. H. Klösgen R. B. (2011). Induction of stromules formation by extracellular sucrose and glucose in epidermal leaf tissue of *Arabidopsis thaliana*. BMC Plant Biol. 11:115 10.1186/1471-2229-11-115 21846357 PMC3167769

[B176] Schattat M. H. Klösgen R. B. Mathur J. (2012b). New insights on stromules: stroma filled tubules extended by independent plastids. Plant Signal. Behav. 7, 1132–1137. 10.4161/psb.21342 22899053 PMC3489645

[B177] Schimper A. F. W. (1882). Ueber die Gestalten der Stärkebildner und Farbkörper. Bot. Zentralbl. Ref. Organ. Gesamtgeb. Bot. 12, 175–178.

[B178] Schimper A. F. W. (1883). Ueber die Entwickelung der Chlorophyllkörner und Farbkörper. Bot. Z. 41, 105–162.

[B179] Schmidt P. (1870). Ueber Einige Wirkungen des Lichts auf Pflanzen. Breslau: Robert Nischkowsky.

[B180] Schmidt von Braun S. Schleiff E. (2008). The chloroplast outer membrane protein CHUP1 interacts with actin and profilin. Planta 227, 1151–1159. 10.1007/s00425-007-0688-7 18193273

[B181] Schnurr J. A. Shockey J. M. de Boer G. J. (2002). Fatty acid export from the chloroplast. Molecular characterization of a major plastidial acyl-coenzyme A synthetase from Arabidopsis. Plant Physiol. 129, 1700–1709. 10.1104/pp.003251 12177483 PMC166758

[B182] Schwarzländer M. Fricker M. D. Müller C. Marty L. Brach T. Novak J.. (2008). Confocal imaging of gltathione redox potential in living plant cells. J. Microsc. 231, 299–316. 10.1111/j.1365-2818.2008.02030.x 18778428

[B183] Senn G. (1908). Die Gestalts- und Lageveränderung der Pflanzen- Chromatophoren. Leipzig: Wilhelm Engelmann.

[B184] Seo Y. S. Kim E. Y. Kim J. H. Kim W. T. (2009). Enzymatic characterization of class I DAD1-like acylhydrolase members targeted to chloroplast in Arabidopsis. FEBS Lett. 583, 2301–2307. 10.1016/j.febslet.2009.06.021 19527719

[B185] Shanmugabalaji V. Besagni C. Piller L. E. Douet V. Ruf S. Bock R.. (2013). Dual targeting of a mature plastoglobulin/fibrillin fusion protein to chloroplast plastoglobules and thylakoids in transplastomic tobacco plants. Plant Mol. Biol. 81, 13–25. 10.1007/s11103-012-9977-z 23086498

[B186] Shaw D. J. Gray J. C. (2011). Visualisation of stromules in transgenic wheat expressing a plastid-targeted yellow fluorescent protein. Planta 233, 961–970. 10.1007/s00425-011-1351-x 21274561

[B187] Shumskaya M. Bradbury L. M. Monaco R. R. Wurtzel E. T. (2012). Plastid localization of the key carotenoid enzyme phytoene synthase is altered by isozyme, allelic variation, and activity. Plant Cell 24, 3725–3741. 10.1105/tpc.112.104174 23023170 PMC3480298

[B188] Sidorov V. A. Kasten D. Pang S. Z. Hajdukiewicz P. T. Staub J. M. Nehra N. S. (1999). Technical advance: stable chloroplast transformation in potato: use of green fluorescent protein as a plastid marker. Plant J. 19, 209–216. 10.1046/j.1365-313X.1999.00508.x 10476068

[B189] Smith S. M. Fulton D. C. Chia T. Thorneycroft D. Chapple A. Dustan H.. (2004). Diurnal changes in the transcriptome encoding enzymes of starch metabolism provide evidence for both transcriptional and posttranscriptional regulation of starch metabolism in Arabidopsis leaves. Plant Physiol. 136, 2687–2699. 10.1104/pp.104.044347 15347792 PMC523333

[B190] Sokolov L. N. Dominguez-Solis J. R. Allary A. L. Buchanan B. B. Luan S. (2006). A redox-regulated chloroplast protein phosphatase binds to starch diurnally and functions in its accumulation. Proc. Natl. Acad. Sci. U.S.A. 103, 9732–9737. 10.1073/pnas.0603329103 16772378 PMC1480475

[B191] Stael S. Kmiecik P. Willems P. van der Kelen K. Coll N. S. Teige M.. (2014). Plant innate immunity - sunny side up? Trends Plant Sci. 20, 3–11. 10.1016/j.tplants.2014.10.002 25457110 PMC4817832

[B192] Sugimoto H. Kusumi K. Noguchi K. Yano M. Yoshimura A. Iba K. (2007). The rice nuclear gene, VIRESCENT 2, is essential for chloroplast development and encodes a novel type of guanylate kinase targeted to plastids and mitochondria. Plant J. 52, 512–527. 10.1111/j.1365-313X.2007.03251.x 17727616

[B193] Sugiura K. Nagai T. Nakano M. Ichinose H. Nakabayashi T. Ohta N.. (2015). Redox sensor proteins for highly sensitive direct imaging of intracellular redox state. Biochem. Biophys. Res. Commun. 457, 242–248. 10.1016/j.bbrc.2014.12.095 25592971

[B194] Sun X. Ling S. Lu Z. Ouyang Y. D. Liu S. Yao J. (2014). OsNF-YB1, a rice endosperm-specific gene, is essential for cell proliferation in endosperm development. Gene 551, 214–221. 10.1016/j.gene.2014.08.059 25178525

[B195] Szydlowski N. Ragel P. Raynaud S. Lucas M. M. Roldan I. Montero M.. (2009). Starch granule initiation in Arabidopsis requires the presence of either class IV or class III starch synthases. Plant Cell 21, 2443–2457. 10.1105/tpc.109.066522 19666739 PMC2751949

[B196] Taiz L. Zeiger E. Møller I. M. Murphy A. (2015). Plant Physiology and Development, 6th Edn. Sunderland, MA: Sinauer Associates Inc.

[B197] Takahashi S. Badger M. R. (2011). Photoprotection in plants: a new light on photosystem II damage. Trends Plant Sci. 16, 53–60. 10.1016/j.tplants.2010.10.001 21050798

[B198] Tan X. Wang Q. Tian B. Zhang H. Lu D. Zhou J. (2011). A Brassica napus lipase locates at the membrane contact sites involved in chloroplast development. PLoS ONE 6:e26831. 10.1371/journal.pone.0026831 22046373 PMC3202582

[B199] Teardo E. Formentin E. Segalla A. Giacometti M. Marin O. Zanetti M.. (2011). Dual localization of plant glutamate receptor AtGLR3.4 to plastids and plasmamembrane. BBA Bioenerget. 1807, 359–367. 10.1016/j.bbabio.2010.11.008 21110940

[B200] Teng Y. S. Su Y. S. Chen L. J. Lee Y. J. Hwang I. Li H. M. (2006). Tic21 is an essential translocon component for protein translocation across the chloroplast inner envelope membrane. Plant Cell 18, 2247–2257. 10.1105/tpc.106.044305 16891400 PMC1560916

[B201] Terasawa K. Sato N. (2005). Visualization of plastid nucleoids *in situ* using the PEND–GFP fusion protein. Plant Cell Physiol. 46, 649–660. 10.1093/pcp/pci070 15746158

[B202] Tirlapur U. K. Dahse I. Reiss B. Meurer J. Oelmuller R. (1999). Characterization of the activity of a plastid-targeted green fluorescent protein in Arabidopsis. Eur. J. Cell Biol. 78, 233– 240. 10.1016/S0171-9335(99)80056-9 10350211

[B203] Vidi P. A. Kanwischer M. Baginsky S. Austin J. R. Csucs G. Dörmann P.. (2006). Tocopherol cyclase (VTE1) localization and vitamin E accumulation in chloroplast plastoglobule lipoprotein particles. J. Biol. Chem. 281, 11225–11234. 10.1074/jbc.M511939200 16414959

[B204] Villarejo A. Burén S. Larsson S. Déjardin A. Monné M. Rudhe C.. (2005). Evidence for a protein transported through the secretory pathway en route to the higher plant chloroplast. Nat. Cell Biol. 7, 1224–1231. 10.1038/ncb1330 16284624

[B205] Vitha S. McAndrew R. S. Osteryoung K. W. (2001). FtsZ ring formation at the chloroplast division site in plants. J. Cell Biol. 153, 111–120. 10.1083/jcb.153.1.111 11285278 PMC2185535

[B206] Vladimirou E. Li M. Aldridge C. P. Frigerio L. Kirkilionis M. Robinson C. (2009). Diffusion of a membrane protein, Tat subunit Hcf106, is highly restricted within the chloroplast thylakoid network. FEBS Lett. 583, 3690–3696. 10.1016/j.febslet.2009.10.057 19854178

[B207] von Braun S. S. Sabetti A. Hanic-Joyce P. J. Gu J. Schleiff E. Joyce P. B. M. (2007). Dual targeting of the tRNA nucleotidyltransferase in plants: not just the signal. J. Exp. Bot. 58, 4083–4093. 10.1093/jxb/erm267 18182422

[B208] Wada M. (2013). Chloroplast movement. Plant Sci. 210, 177–182. 10.1016/j.plantsci.2013.05.016 23849124

[B209] Wang Q. Sullivan R. W. Kight A. Henry R. L. Huang J. Jones A. M.. (2004). Deletion of the chloroplast-localized Thylakoid formation1 gene product in Arabidopsis leads to deficient thylakoid formation and variegated leaves. Plant Physiol. 136, 3594–3604. 10.1104/pp.104.049841 15516501 PMC527158

[B210] Wang Z. Pesacreta T. C. (2004). A subclass of myosin XI is associated with mitochondria, plastids, and the molecular chaperone subunit TCP-1α in maize. Cell Motil. Cytoskel. 57, 218–232. 10.1002/cm.10168 14752806

[B211] Waters M. T. Fray R. G. Pyke K. A. (2004). Stromule formation is dependent upon plastid size, plastid differentiation status and the density of plastids within the cell. Plant J. 39, 655–667. 10.1111/j.1365-313X.2004.02164.x 15272881

[B212] Whatley J. M. McLean B. Juniper B. E. (1991). Continuity of chloroplast and endoplasmic-reticu- lum membranes in Phaseolus vulgaris. New Phytol. 117, 209–217. 10.1111/j.1469-8137.1991.tb04901.x

[B213] Wiedenmann J. Ivanchenko S. Oswald F. Schmitt F. Röcker C. Salih A.. (2004). EosFP, a fluorescent marker protein with UV-inducible green-to-red fluorescence conversion. Proc. Natl. Acad. Sci. U.S.A. 101, 15905–15910. 10.1073/pnas.0403668101 15505211 PMC528746

[B214] Wildman S. G. Hongladarom T. Honda S. I. (1962). Chloroplasts and mitochondria in living plant cells: cinephotomicrographic studies. Science 138, 434–436. 10.1126/science.138.3538.434 17794918

[B215] Wise R. R. (2007). The diversity of plastid form and function. chapter 1, in The Structure and Function of Plastids, eds Wise R. R. Hoober J. K. (Dordrecht: Springer), 3–26.

[B216] Wooding F. B. P. Northcot D. (1965). Association of endo- plasmic reticulum and plastids in acer and pinus. Am. J. Bot. 52, 526–529. 10.2307/2440270

[B217] Wu Q. Luo A. Zadrozny T. Sylvester A. Jackson D. (2013). Fluorescent protein marker lines in maize: generation and applications. Int. J. Dev. Biol. 57, 535–543. 10.1387/ijdb.130240qw 24166436

[B218] Xu C. C. Fan J. L. Cornish A. J. Benning C. (2008). Lipid trafficking between the endoplasmic reticulum and the plastid in Arabidopsis requires the extraplastidic tgd4 protein. Plant Cell 20, 2190–2204. 10.1105/tpc.108.061176 18689504 PMC2553622

[B219] Xu C. C. Moellering E. R. Muthan B. Fan J. L. Benning C. (2010). Lipid transport mediated by Arabidopsis tgd proteins is unidirectional from the endoplas- mic reticulum to the plastid. Plant Cell Physiol. 51, 1019–1028. 10.1093/pcp/pcq053 20410050

[B220] Yagi Y. Ishizaki Y. Nakahira Y. Tozawa Y. Shiina T. (2012). Eukaryotic-type plastid nucleoid protein pTAC3 is essential for transcription by the bacterial-type plastid RNA polymerase. Proc. Natl. Acad. Sci. U.S.A. 109, 7541–7546. 10.1073/pnas.1119403109 22529394 PMC3358912

[B221] Yamane K. Mitsuya S. Taniguchi M. Miyake H. (2012). Salt−induced chloroplast protrusion is the process of exclusion of ribulose−1, 5−bisphosphate carboxylase/oxygenase from chloroplasts into cytoplasm in leaves of rice. Plant Cell Environ. 35, 1663–1671. 10.1111/j.1365-3040.2012.02516.x 22489666

[B222] Yang Y. Jin H. Chen Y. Lin W. Wang C. Chen Z.. (2012). A chloroplast envelope membrane protein containing a putative LrgB domain related to the control of bacterial death and lysis is required for chloroplast development in *Arabidopsis thaliana*. New Phytol. 193, 81–95. 10.1111/j.1469-8137.2011.03867.x 21916894

[B223] Ytterberg A. J. Peltier J. van Wijk K. J. (2006). Protein profiling of plastoglobules in chloroplasts and chromoplasts. A surprising site for differential accumulation of metabolic enzymes. Plant Physiol. 140, 984–997. 10.1104/pp.105.076083 16461379 PMC1400577

[B224] Zeeman S. C. Kossmann J. Smith A. M. (2010). Starch: its metabolism, evolution and biotechnological modification in plants. Annu. Rev. Plant Biol. 61, 209–234. 10.1146/annurev-arplant-042809-112301 20192737

[B225] Zentgraf U. (2007). Oxidative stress and leaf senescence, in Senescence Processes in Plants. Annual Plant Reviews, Vol. 26, ed Gan S. (Oxford, UK: Blackwell Publishing Ltd.), 69–86.

[B226] Zhang L. Ren Y. Lu B. Yang C. Feng Z. Liu Z.. (2015). FLOURY ENDOSPERM7 encodes a regulator of starch synthesis and amyloplast development essential for peripheral endosperm development in rice. J. Exp. Bot. pii, erv469. 10.1093/jxb/erv469 26608643 PMC4737065

[B227] Zhang X. Hu J. (2010). The *Arabidopsis* chloroplast division protein DYNAMIN-RELATED PROTEIN5B also mediates peroxisome division. Plant Cell 22, 431–442. 10.1105/tpc.109.071324 20179140 PMC2845408

